# Transcriptional profiling unveils molecular subgroups of adaptive and maladaptive right ventricular remodeling in pulmonary hypertension

**DOI:** 10.1038/s44161-023-00338-3

**Published:** 2023-09-28

**Authors:** Fatemeh Khassafi, Prakash Chelladurai, Chanil Valasarajan, Sreenath Reddy Nayakanti, Sandra Martineau, Natascha Sommer, Tetsuro Yokokawa, Olivier Boucherat, Aryan Kamal, David G. Kiely, Andrew J. Swift, Samer Alabed, Junichi Omura, Sandra Breuils-Bonnet, Carsten Kuenne, Francois Potus, Stefan Günther, Rajkumar Savai, Werner Seeger, Mario Looso, Allan Lawrie, Judith B. Zaugg, Khodr Tello, Steeve Provencher, Sébastien Bonnet, Soni Savai Pullamsetti

**Affiliations:** 1 Max Planck Institute for Heart and Lung Research, Bad Nauheim, Germany; 2 Department of Internal Medicine, Universities of Giessen and Marburg Lung Center (UGMLC), Member of the German Center for Lung Research (DZL), Excellence Cluster Cardio-Pulmonary Institute (CPI), Justus-Liebig University, Giessen, Germany; 3 Pulmonary Hypertension and Vascular Biology Research Group of Quebec Heart and Lung Institute, Department of Medicine, Laval University, Quebec, Canada; 4 Institute for Lung Health (ILH), Justus-Liebig University, Giessen, Germany; 5 Department of Cardiovascular Medicine, Fukushima Medical University, Fukushima, Japan; 6 European Molecular Biology Laboratory, Heidelberg, Germany; 7 Department of Infection, Immunity and Cardiovascular Disease, University of Sheffield, Sheffield, UK; 8 Sheffield Pulmonary Vascular Disease Unit, Royal Hallamshire Hospital, Sheffield Teaching Hospitals NHS Foundation Trust, Sheffield, UK; 9 NIHR Biomedical Research Center, Sheffield, UK; 10 National Heart and Lung Institute, Imperial College London, London, UK

**Keywords:** Cardiac hypertrophy, Predictive markers

## Abstract

Right ventricular (RV) function is critical to prognosis in all forms of pulmonary hypertension. Here we perform molecular phenotyping of RV remodeling by transcriptome analysis of RV tissue obtained from 40 individuals, and two animal models of RV dysfunction of both sexes. Our unsupervised clustering analysis identified ‘early’ and ‘late’ subgroups within compensated and decompensated states, characterized by the expression of distinct signaling pathways, while fatty acid metabolism and estrogen response appeared to underlie sex-specific differences in RV adaptation. The circulating levels of several extracellular matrix proteins deregulated in decompensated RV subgroups were assessed in two independent cohorts of individuals with pulmonary arterial hypertension, revealing that NID1, C1QTNF1 and CRTAC1 predicted the development of a maladaptive RV state, as defined by magnetic resonance imaging parameters, and were associated with worse clinical outcomes. Our study provides a resource for subphenotyping RV states, identifying state-specific biomarkers, and potential therapeutic targets for RV dysfunction.

## Abstract (web summary)

Khassafi et al. identify molecular subgroups of right ventricular (RV) dysfunction using transcriptomic analysis of RV samples from individuals with compensated RV hypertrophy or decompensated RV failure and two rat models of RV dysfunction. Several extracellular matrix genes found to be deregulated in decompensated RV subgroups were validated at the protein level in two independent cohorts of individuals with pulmonary arterial hypertension, revealing their predictive biomarker potential in maladaptive RV development.

## Main

RV dysfunction can occur as a consequence of a variety of underlying clinical conditions that cause pressure overload, volume overload or intrinsic cardiomyopathies^1^. A major pathological mechanism of RV failure with normal left heart function is increased afterload due to pulmonary hypertension (PH)^2^. As progressive RV dysfunction is associated with increased morbidity and mortality, accurate assessment and characterization of RV function and molecular phenotype in patients with PH is crucial in diagnosis and management of the disease^1^.

The normal RV structure consists of a compliant thin wall that accommodates substantial changes in volume (preload), and therefore an intrinsic capacity to adapt to progressive increases in afterload caused by gradual elevation in pulmonary arterial pressures as is typically observed in PH^3^. Persistently elevated afterload caused by pulmonary vascular remodeling activates adaptive RV alterations. However, the critical factors that mediate deterioration from adaptive to maladaptive hypertrophy and RV failure remain elusive. This is particularly important when adaptive remodeling fails to maintain the stroke volume (SV), which usually causes RV dilatation and failure^4^.

A deeper understanding of the molecular mechanisms governing adaptive responses and their transition to RV failure has recently been explored, by several omics analyses in human and animal models of RV dysfunction, which revealed molecular mechanisms associated with adverse RV remodeling^5–14^. Of note, Park et al. compared the RV transcriptome from two rat models of PH and identified the common signature of RV remodeling between monocrotaline (MCT) and Sugen5416/hypoxia (SuHx) rats and validated some of the epithelial-to-mesenchymal transition (EMT)-associated target genes in human RV tissue^5^. In addition, Kobayashi et al. investigated transcriptome and metabolome changes in SuHx rat models after chrysin treatment compared with control RV samples^6^. Moreover, several proteomic profiling studies of RV in various PH animal models have been generated^7–10^. Hindmarch et al. performed an integrative transcriptome and proteome analysis of the MCT rat model, in which they identified 410 proteins associated with the RV failure phenotype^7^. In addition, a human RV proteome was recently published by Boucherat et al., which showed the altered protein profile associated with RV remodeling in pulmonary arterial hypertension (PAH) by an integrative transcriptome and proteomics analysis and also validated a panel of proteins in animal models of PH^11^. Recent transcriptomic and proteomic studies of plasma from various human PAH cohorts have been performed^12–14^, in which various proteins in the participants’ blood were identified and their correlation with prognosis or survival in PAH has been demonstrated.

However, downstream mechanisms that drive adaptive remodeling to RV failure have not yet been investigated. The progression from initial stages into the uncoupled failing stage is a continuum in which the remodeling events overlap and smoothly transit throughout the stages^15^. This leads to the assumption that there may be different phases of adaptive and maladaptive remodeling. On the other hand, RV dysfunction needs clinically different treatments as the RV is supported differently depending on the stage of remodeling. Hence, it is important to develop accurate diagnostic tools that can assess RV disease severity and detect early maladaptive changes in RV function and structure^16–19^.

In this Article, we aim to (i) perform precise molecular phenotyping of RV remodeling by identifying subgroups within compensated and decompensated states of RV function in individuals with PH and animal models of RV dysfunction, (ii) investigate sex-specific transcriptional differences in RV remodeling, and (iii) evaluate whether a number of potential RV-relevant biomarkers could distinguish early- and late-decompensated states in individuals with PAH, and their impact on clinical features and prognosis.

## Results

### Transcriptomic analysis of the right ventricle in two rat models of pulmonary hypertension

**Extended Data Fig. 1:**
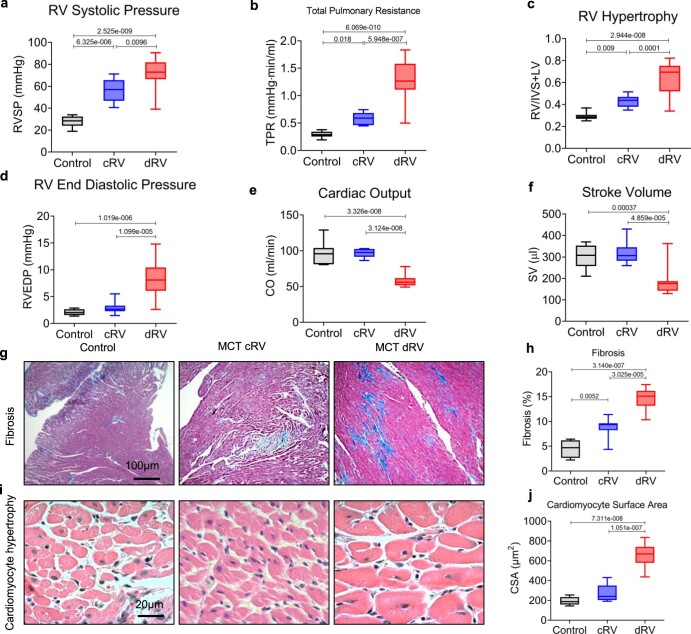
Hemodynamic assessment and RV function in MCT-induced PH rats. Hemodynamic data collected by closed-chest right heart catheterization. RV hypertrophy was calculated as Fulton index (weight ratio of RV and (LV + septum)). (**a**) RV systolic pressure (RVSP), (**b**) total pulmonary resistance (TPR) (**c**) RV hypertrophy, (**d**) RV end-diastolic pressure (RVEDP), (**e**) cardiac output (CO). (**f**) Stroke volume (SV). Number of samples (n) in each group of comparison= 10. (**g**) Representative images of RV stained with Masson’s trichrome from control and MCT-treated rats with compensated RV (cRV) and decompensated RV (dRV) states. (**h**) Quantification of cardiac fibrosis in the same rat samples. (**i**) Representative images of RVs stained with H&E from control and MCT-treated rats. (**j**) Quantification of cardiomyocyte cross-sectional area (CSA) representing cardiomyocyte hypertrophy (from the same rat samples). (**h**, **j**) n(control)=4, n(compensated) = 9, n(decompensated)=9. Data are presented as mean ± SEM. (P value has been calculated by one-way ANOVA followed by Tukey’s multiple comparisons test). In all the box plots: Central bands represents 50% quantile (median), box interquartile ranges: 25–75%, and whiskers set to max/min, 1.5 IQR above/below the box. Source data

**Extended Data Fig. 2:**
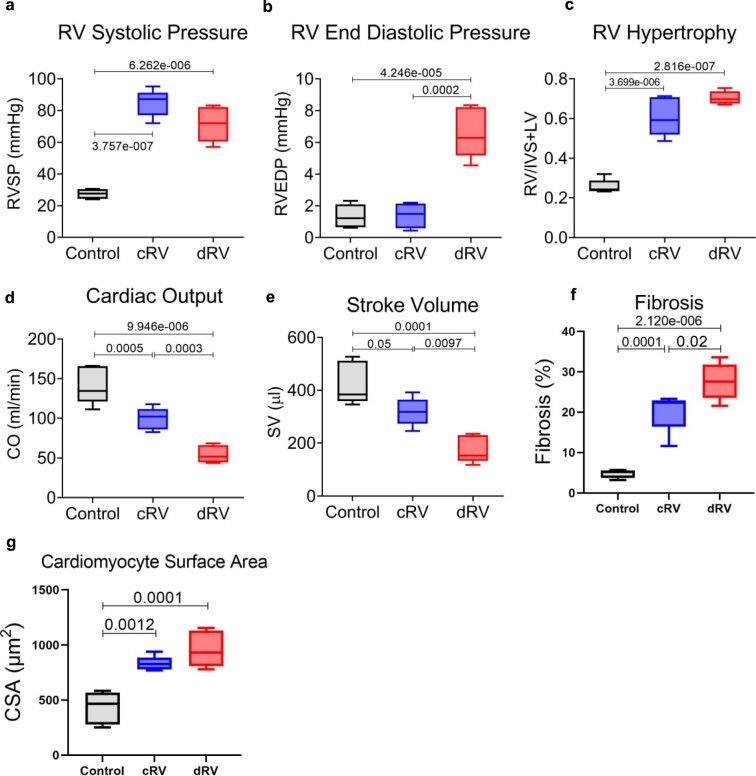
Hemodynamic assessment and RV function in rat PAB model. (**a**) RV systolic pressure (RVSP), (**b**) RV end-diastolic pressure (RVEDP), (**c**) RV hypertrophy (RVH, calculated as Fulton index (weight ratio of RV to LV + septum)), (**d**) cardiac output (CO), (**e**) stroke volume (SV), (**f**) representative quantification of cardiac fibrosis from the PAB-rats RV samples stained with Masson’s trichrome for control, compensated RV (cRV) and decompensated RV (dRV) states. (**g**) Representative quantification of H&E staining for cardiomyocyte cross-sectional area (CSA) representing cardiomyocyte hypertrophy in the same RV samples. Data are presented as mean ± SEM. (P value has been calculated by one-way ANOVA followed by Tukey’s multiple comparisons test). In all the box plots: Central bands represents 50% quantile (median), box interquartile ranges: 25–75%, and whiskers set to max/min, 1.5 IQR above/below the box. Number of samples (n) in each group of comparison=5. PAB-induced PH has been evaluated by significant elevations of RVSP, TPR, and RV hypertrophy in compensated RV state, while RVEDP was distinctly altered in decompensated RV, which along with clinical RV failure signs enabled us to clearly distinguish the decompensated from compensated RV condition in PAB rats. However, cardiac output (CO), as well as stroke Volume (SV) were continuously reduced in PAB rats unlike the MCT model. Source data

To investigate the transcriptional comparison between compensated and decompensated RV tissues affected by increased afterload in PH, we first classified the pathophysiology of RV function into normal, compensated hypertrophy and decompensated failure, based on RV function and hemodynamic parameters, in two rodent models of PH and RV dysfunction, that is, in models induced by MCT (Extended Data Fig. 1a–f and Methods) and pulmonary artery banding (PAB) in rats (Extended Data Fig. 2a–f and Methods).

**Fig. 1:**
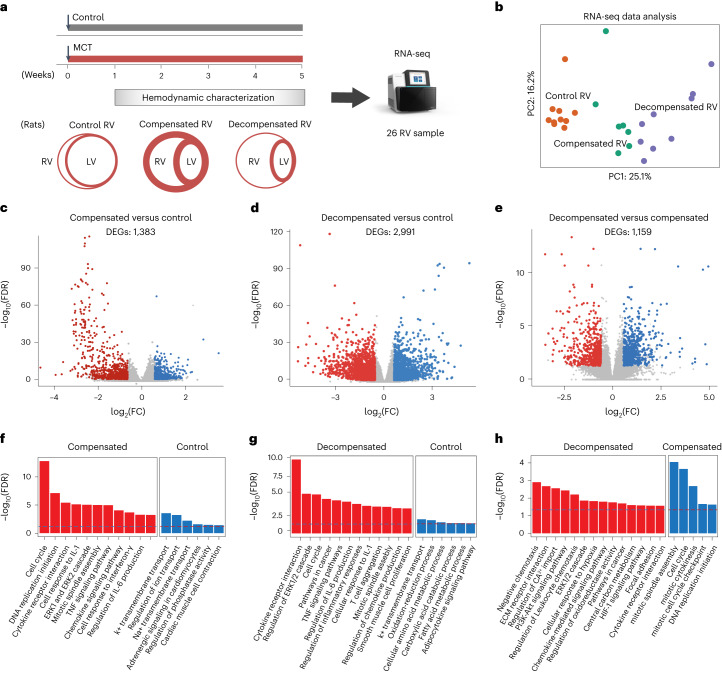
Transcriptomic analysis of right ventricle in a rat model of MCT-induced pulmonary hypertension. **a**, Experimental design for the hemodynamic and transcriptomic characterization of RV samples in control (normal), compensated and decompensated states from MCT-induced PH rats. **b**, PC analysis was performed on the normalized RNA-seq data to visualize the gene expression profiles of all the RV samples. Control RV samples are shown in orange, compensated RV samples in green, and decompensated RV samples in purple. **c**–**e**, Volcano plots show the significance of each expressed gene (−log_10_ false discovery rate (FDR) values on the *y* axis), plotted against the logarithmic fold change (log_2_FC values on the *x* axis), in each pair of comparisons. DEGs were identified by DESeq2 (base mean expression ≥ 5; −0.585 ≤ log_2_FC ≥ 0.585; FDR ≤ 0.05). **c**, Compensated (red) versus control RV (blue) (**c**), decompensated (red) versus control RV (blue) (**d**) and decompensated (red) versus compensated RV (blue) (**e**). **f**–**h**, Top-selected differentially enriched pathways for each pair of comparisons. Compensated versus control RV (**f**), decompensated versus control RV (**g**) and decompensated versus compensated RV (**h**). The dashed line shows FDR = 0.05.

RNA sequencing (RNA-seq) was performed on RV tissues obtained from 30 rats, grouped into control, compensated and decompensated RV samples from MCT-induced PH rats (Fig. 1a). Principle component (PC) analysis demonstrated a clear separation between the three groups through PC1 (Fig. 1b). Analysis of differentially expressed genes (DEGs) revealed substantial transcriptional changes in compensated (1,383 genes) and decompensated (2,991 genes) RV samples, compared with control RV samples, as well as 1,159 genes differentially expressed between decompensated and compensated RV samples (Fig. 1c–e and Supplementary Data 1). The correlation between the samples based on their gene expression profile and top 50 significant DEGs for each pair of comparison shown in Supplementary Fig. 1. Pathway enrichment analysis revealed that the DEGs in compensated RV samples, compared to controls, were associated with the cell cycle, DNA replication initiation, response to interleukin-1 and cytokine-mediated signaling pathways, while genes associated with extracellular matrix (ECM) organization, the immune responses, cardiac conduction and different metabolic processes were differentially regulated in decompensated RV samples (Fig. 1f, g). Regarding the transition of adaptive RV to maladaptive RV failure, genes associated with response to hypoxia, ECM receptor interactions and cell adhesion were notably dysregulated in decompensated RV samples, compared with compensated RV samples (Fig. 1h and Supplementary Fig. 2).

**Fig. 2:**
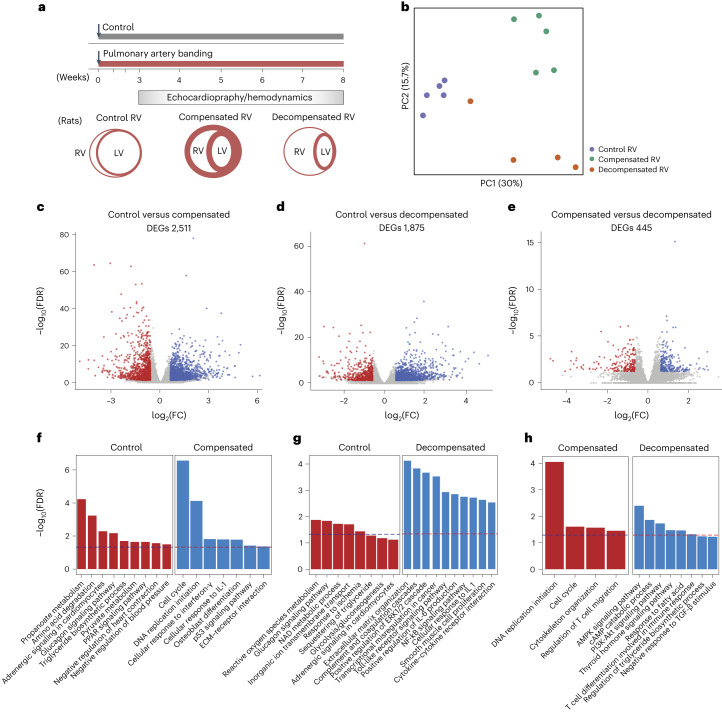
Transcriptomic analysis of the right ventricle in a rat model of pulmonary artery banding. **a**, Experimental design for the hemodynamic and transcriptomic characterization of RV samples in control (normal), compensated and decompensated states from rats subjected to PAB. **b**, PC analysis was performed on normalized RNA-seq data to visualize the gene expression profiles of all the RV samples. Control RV samples are shown in purple, compensated RV samples in green, and decompensated RV samples in orange. **c**–**e**, Volcano plots show the significance of each expressed gene (−log_10_ FDR values on the *y* axis), plotted against the logarithmic fold change (log_2_FC values on the *x* axis), in each pair of comparisons. DEGs were identified by DESeq2 (base mean expression ≥ 5; −0.585 ≤ log_2_FC ≥ 0.585; FDR ≤ 0.05). (**c**) Compensated (blue) versus control RV (red) (**c**), decompensated (blue) versus control RV (red) (**d**) and decompensated (blue) versus compensated RV (red) (**e**). **f**–**h**, Top selected differentially enriched pathways for each pair of comparisons. Compensated versus control RV (**f**), decompensated versus control RV (**g**) and decompensated versus compensated RV (**h**). The dashed line shows FDR = 0.05.

Furthermore, RNA-seq analysis of 15 RV tissue samples obtained from PAB rats, including 5 samples in each control, compensated and decompensated groups, was performed (Fig. 2a). PC analysis separated samples from control, compensated and decompensated PAB RV samples while indicating a large heterogeneity in decompensated RV samples (Fig. 2b and Supplementary Fig. 3a). DEG analysis revealed dysregulation of 2,511 and 1,875 genes in compensated and decompensated RV groups respectively, compared with control, while lower DEGs (445) between compensated and decompensated RV samples suggest less distinct profiles of decompensation in PAB rats compared with MCT-treated rats (Fig. 2c–e and Supplementary Data 2). Correlation between the samples, the top 50 significant DEGs and enriched GO and KEGG pathways in all three contrasts are provided in Supplementary Figs. 3 and 4. Similarly to the MCT model, the enrichment analysis of DEGs of compensated RV samples revealed cell cycle, DNA replication initiation and increased response to interleukin-1 and interferon-γ. However, downregulation of a few other pathways such as amino acids, pyruvate and propanoate metabolism was also distinctly regulated in PAB-associated compensated RV samples (Fig. 2f). In contrast, ECM reorganization, nuclear factor (NF)-kB signaling pathway, various immune-related cytokines such as tumor necrosis factor (TNF), along with hypoxia and leukocyte recruitment were significantly increased in the the PAB rat decompensated phase, which was very similar to the dysregulated pattern observed in the MCT rat decompensated RV samples (Fig. 2g and Supplementary Data 2). DEGs in decompensated versus compensated RV samples in PAB rats were mostly associated with upregulation of the AMPK signaling pathway, cAMP catabolism and the PI3K–Akt signaling pathway, and downregulation of mitotic proliferation (Fig. 2h).

### Transcriptomic analysis of human adaptive and maladaptive right ventricle

**Fig. 3:**
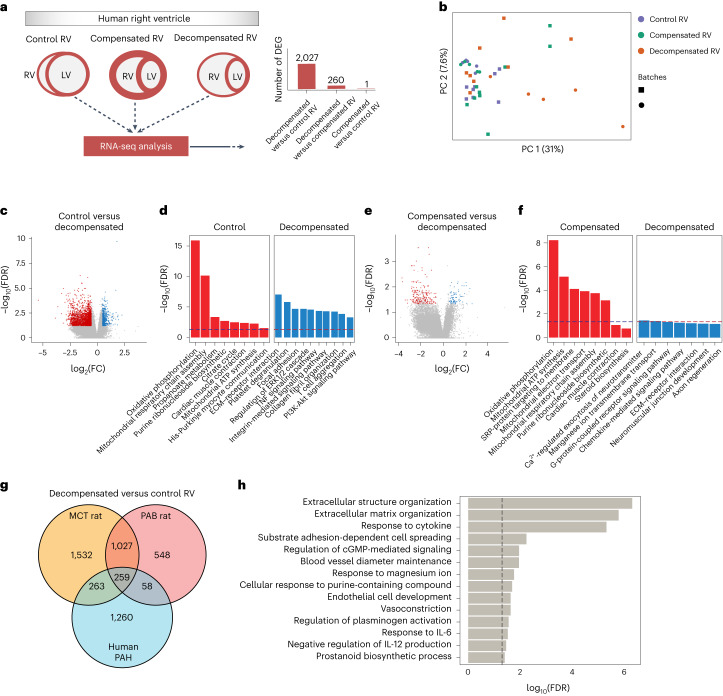
Transcriptomic analysis of adaptive and maladaptive remodeling in the human right ventricle. **a**, RNA-seq was performed on human RV tissues that were clinically classified by hemodynamic assessment and clinical symptoms into control/normal (*n* = 13), compensated (*n* = 14) and decompensated (*n* = 13) RV states, obtained and sequenced in two batches, underwent standard quality-control assessment, batch effect removal, and normalization. DEGs were identified by DESeq2 (base mean expression ≥ 5; −0.585 ≤ log_2_FC ≥ 0.585; FDR ≤ 0.05). **b**, PC analysis on the normalized/batch-corrected RNA-seq data from 40 human RV samples. Colors distinguish different RV states, and shapes show two different batches of data. **c**, Volcano plot highlighting the significant DEGs (−0.585 ≤ log_2_FC ≥ 0.585 and FDR ≤ 0.05 in decompensated (blue) versus normal RV (red). **d**, Top-selected pathways enriched for decompensated versus normal RV samples. **e**, Volcano plot highlighting the significant DEGs in decompensated (blue) versus compensated RV (red) samples. **f**, Top-selected pathways enriched for decompensated versus compensated RV samples. The dashed line shows FDR = 0.05. **g**, Common and distinct DEGs for decompensated RV versus normal RV samples in three transcriptome datasets displayed in the Venn diagram. Orange, MCT rat; red, PAB rat; blue, human decompensated RV versus normal. **h**, Top significantly enriched pathways for 259 common DEGs of decompensated versus normal samples between all three datasets. The dashed line shows FDR = 0.05. Source data

To investigate the underlying molecular signature of RV dysfunction in humans, RNA-seq was performed on 40 human RV tissues that were classified, by hemodynamic assessment and clinical symptoms^20^, into: control, compensated RV and decompensated RV states (Fig. 3a and Methods). A batch correction method was applied before any downstream analysis as the human RV samples were obtained in two different time points. PC analysis showed that RV samples were separated mostly based on the different RV states rather than different batches of sampling (Fig. 3b and Supplementary Fig. 5a). However, a substantial heterogeneity existed, especially within the samples. Differential expression analysis showed dysregulation of 2,027 genes in decompensated RV samples, compared with control RV samples, and 260 DEGs between decompensated and compensated RV samples. We did not detect any significant DEGs associated with compensated RV samples compared to controls, which could be due to the molecular heterogeneity within the normal and hypertrophic RV samples or the functional similarities between them. Enrichment analysis showed that DEGs in the decompensated RV samples were significantly associated with increased ECM and receptor interaction and cell adhesion as well as activation of cytokines such as TNF. In contrast, pathways associated with the tricarboxylic acid (TCA) cycle and cardiac muscle contraction were significantly downregulated in decompensated RV samples, while they were upregulated in the compensated RV state (Fig. 3c–f).

Finally, we looked for similarities between the DEG results of all datasets. As expected, we observed higher similarity between two rat models, in terms of decompensated versus control RV (1,286 genes), while in terms of similarity with human, more common genes were regulated between MCT-induced decompensated and human PAH decompensated RV samples as compared to PAB (522 versus 317 genes; Fig. 3g). Both gene sets were significantly enriched for ECM and integral membrane proteins; however, TNF regulation of cell death seemed to be regulated in MCT-induced RV samples, similar to human decompensated RV samples, while not in the PAB model, which suggests more molecular similarity of the MCT model to the human RV remodeling. Further, focusing on 259 genes that were commonly regulated in decompensated RV samples in all three datasets (Fig. 3g), we found that ECM along with the response to several cytokines as well as vasoconstriction and vasodilation mechanisms were commonly upregulated in all three profiles (Fig. 3h).

### Sex differences influence the molecular signature of right ventricular remodeling

**Fig. 4:**
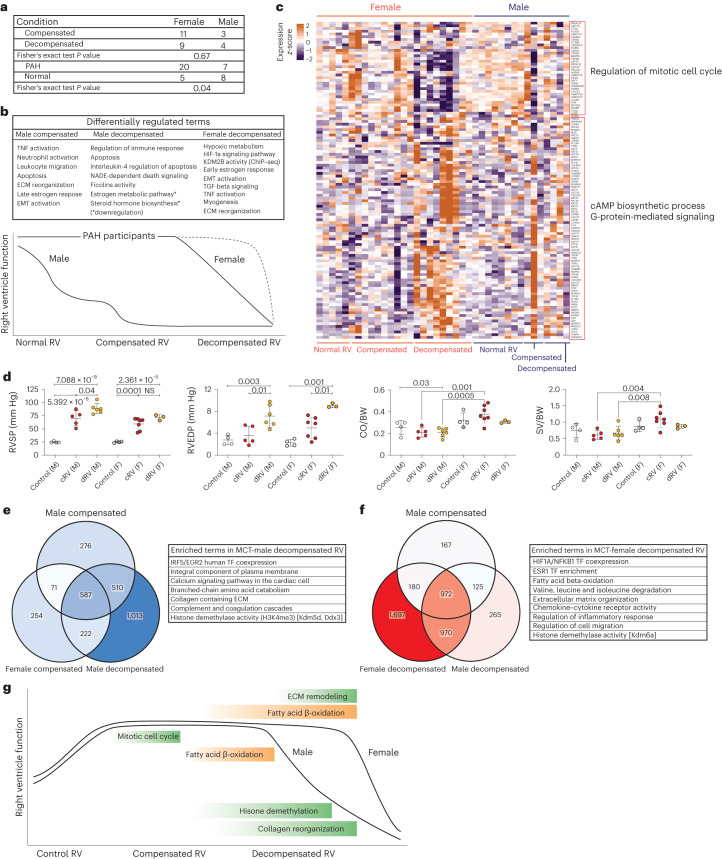
Effects of sex difference on human and MCT rat right ventricular remodeling associated with pulmonary hypertension. **a**, Number of patients in each group, separated by sex, and the Fisher’s exact test *P* value for compensated-versus-decompensated RV and normal-versus-PAH RV pairs. **b**, Schematic of RV dysfunction in male and female participants, which shows a different route (longer) for female decompensated RV failure compared with males. Differentially enriched pathways in male compensated and decompensated RV samples and female decompensated RV demonstrate independent biological routes of maladaptive RV remodeling in female and male PAH participants. **c**, Regulation of 116 differentially regulated genes associated with estrogen and progesterone metabolism (FDR < = 0.05) in all human RV samples. *n* (normal RV) = F:5, M:8, *n* (compensated RV) = F:10, M:3, *n* (decompensated RV) = F:9, M:4. Colors represent the scaled gene expression (rows, *z*-score). **d**, Hemodynamics assessment of MCT rats RV functions for male and female animals. *n* (cRV male) = 5, *n* (dRV male) = 6, *n* (cRV female) = 7, *n* (dRV female) = 3, *n* (control F, M) = 4. Data are presented as the mean ± s.e.m. *P* value was calculated by one-way analysis of variance (ANOVA) followed by Tukey’s multiple-comparisons test. **e**,**f**, Genes and pathways that were specifically regulated in male (**e**) and female (**f**) decompensated RV samples, respectively. **g**, Schematic of RV remodeling in MCT-induced rat model, highlighting the main differentially regulated pathways in male and female animals (green, upregulated; orange, downregulated). cRV, compensated right ventricle; dRV, decompensated right ventricle; M, male; F, female; RVSP, right ventricle systolic pressure; CO, cardiac output; BW, body weight; NS, not significant. Source data

Furthermore, to check for the potential differences in terms of transcriptomic signature between the female and male participants, as sex differences are shown to have an impact on RV adaptation and function^21,22^, we investigated whether (i) the inclusion of males and females has an impact on the downstream analysis, and (ii) there are transcriptome differences between male and female participants separately. Although there were more females in the diseased group compared to controls (*P* value = 0.04), sex did not significantly affect the downstream analysis between RV subgroups (Fig. 4a).

Further comparing the molecular differences within the female group, we did not observe significant alteration in compensated RV, while we observed greater changes in decompensated RVs compared with control RV group, such as downregulation of oxidative phosphorylation, upregulation of early estrogen responses, endothelial cell dysfunction, EMT and ECM remodeling pathways, similar to the DEG analysis of the entire group. When we compared similar pairs in male samples, taking the *P* value < 0.05 to determine the DEGs, we found 739 genes dysregulated in the compensated RV group and 38 genes dysregulated in the male decompensated RV group. Enrichment analysis revealed that ECM remodeling, EMT, leukocyte migration and activation and apoptotic regulation occurred in the male compensated RV group, while they were similarly active along with enhanced activation of apoptotic pathways in the decompensated state. This suggests that male participants may develop maladaptive hypertrophy earlier than females (Fig. 4b). Furthermore, upregulation of estrogen-mediated signaling pathways, in female decompensated RVs compared with normal and compensated RVs, suggests that sex hormones influence RV remodeling in female participants (Fig. 4c), and females might take a different route to develop a decompensated state or maintain the compensated state of the RV longer.

We then performed RNA-seq of both male and female RV data from a rat model of MCT-induced PH to see if this also reflects in animal model. Hemodynamics analysis showed higher deterioration of RV function in male MCT rats compared with their female counterparts especially in terms of cardiac output (Fig. 4d). When comparing the compensated RV versus control group in female and male rats separately, we found similar signaling pathways were upregulated such as the mitotic cell cycle. In contrast, comparison of the decompensated RV samples showed differences between female and male groups, despite the similar ECM remodeling. Comparative analysis showed that in male decompensated RV samples, calcium signaling, amino acid metabolism and histone demethylation were upregulated (Fig. 4e), whereas in female decompensated RVs, fatty acid ß-oxidation and various inflammatory responses were exclusively enriched (Fig. 4f). A summary of altered pathways in male and female MCT-induced rats relative to their RV dysfunction suggests that the delayed maladaptive RV remodeling happens in female rats compared to males, similarly to what we observed in human RV hypertrophy and dilation (Fig. 4g).

### Subclassification of decompensated right ventricle in MCT-induced rats

**Fig. 5:**
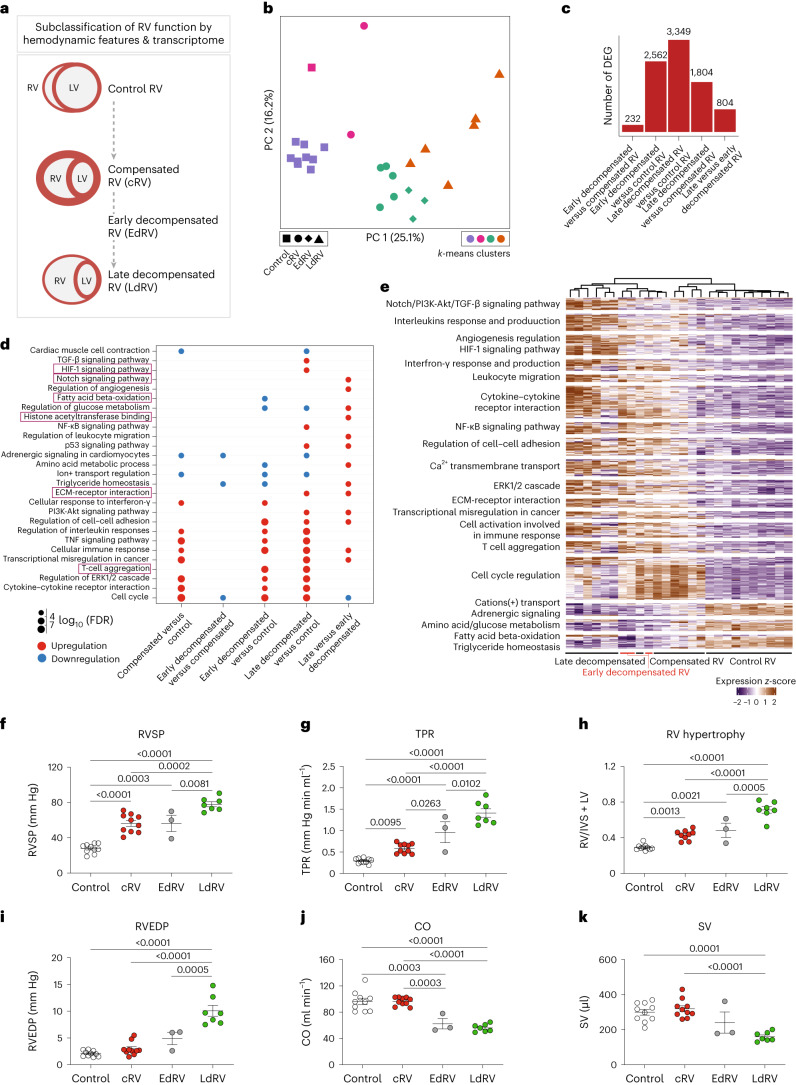
Subclassification of decompensated state based on the transcriptome in MCT-induced pulmonary hypertension. **a**, Schematic of the classification of RV function into normal, compensated and decompensated, along with a further subclassification of the decompensated RV into early and late states, based on both transcriptome and hemodynamic features. **b**, PC analysis was performed on the normalized RNA-seq data, in which the *k*-means clusters are demonstrated by different colors. Three early-decompensated RV samples clustered with the compensated group, while separated from other decompensated samples on both PCs. Different shapes represent different RV states. **c**, The number of DEGs that are significantly regulated in each pairwise comparison (base mean expression ≥ 5, −0.585 ≤ log_2_FC ≥ 0.585, FDR ≤ 0.05). **d**, Cumulative enrichment analysis demonstrating the shortlisted important pathways differentially regulated in each pair of RV subgroups. The size of the dots represents the FDR, the red color shows upregulation, while blue represents downregulation of a pathway in the respective pair. Distinguishing pathways regarding early- to late-decompensated RV transition is highlighted in red. One-sided Fisher’s exact test was used for all the enrichment analysis, which assesses the independent probability of any genes belonging to any set. FDR-corrected *P* value with Benjamini Hochberg method was used for multiple-hypothesis testing. **e**, Scaled gene expression (*z-*score) representation of the 576 cumulative DEGs corresponding to all the altered pathways associated with the transition from compensated to early- and late-decompensated RV in MCT-induced PH. Different groups of biological terms with similar regulation levels are highlighted along the *y* axis. **f**–**k**, Hemodynamic assessment of MCT-induced PH in rats confirmed changes in RV systolic pressure (RVSP; **f**), total pulmonary resistance (TPR; **g**), RV hypertrophy (right ventricular weight to left ventricular plus septal weight ratio; **h**), RV end-diastolic pressure (RVEDP; **i**), cardiac output (CO; **j**) and stroke volume (SV; **k**). *n*(control) = 10, *n*(compensated) = 10, *n*(early decompensated) = 3, *n*(late decompensated) = 7. Data are presented as the mean ± s.e.m. *P* values were calculated by one-way ANOVA followed by Tukey’s multiple-comparisons test. Source data

Although RV transcriptome data from MCT rats were classified into three groups based on hemodynamics, unsupervised clustering (Methods) allowed us to find four molecular clusters in the RV transcriptome of MCT rats (Fig. 5a,b). Using *k*-means clustering, we identified a distinct subgroup of decompensated RV samples that had a similar molecular phenotype to the compensated group, and a distinguished transcriptional signature compared with the rest of decompensated RV samples. We subsequently relabeled these three samples as ‘early-decompensated’ (Fig. 5b) and the other six samples as ‘late-decompensated’ RV samples. DEG analysis of early and late-decompensated subgroups revealed 804 significant DEGs that defined the transition signatures from early- to late-decompensated RV function in the rat model of MCT-induced PH (Fig. 5c).

The transition from compensated RV to early-decompensated RV was associated with a reduction in triglyceride metabolism, fatty acid β-oxidation and cell cycle regulation (Fig. 5d and Supplementary Fig. 6a,b), while a significant increase in NF-κB signaling, ECM and collagen reorganization, as well as active immune cell recruitment and hypoxia response regulation were markedly associated with late-decompensated RV (Fig. 5d and Supplementary Fig. 6c,d). Additional glucose and amino acid metabolism were also observed specifically in late-decompensated RV, which confirms the hypoxic metabolic shift occurs in the transition from early to late stages of RV dysfunction. Figure 5e shows the cumulative differentially regulated genes (*n* = 576) associated with MCT-induced RV remodeling along with their linked pathways.

Based on the transcriptomic analysis confirmation of the transcriptional landscape between compensated and late-decompensated RV states, the hemodynamic data from MCT-induced PH rats were reassessed to validate the early state of decompensation. The transcriptomic status of RV was closer to the compensated RV than to the decompensated RV, which was also consistent with the significant increase in right ventricle systolic pressure (RVSP), right ventricle end-diastolic pressure (RVEDP), total pulmonary resistance and RV hypertrophy that distinguished the early and late states of decompensation; however, the functional shift from the compensated to the early-decompensated RV states was mirrored by a significant reduction in cardiac output (CO) and stroke volume (SV) (Fig. 5f–k). Thus, the combined transcriptomic and hemodynamic data demonstrates the presence of an intermediate early-decompensated phase between the compensated and late-decompensated states in the progressive remodeling of RV in MCT-induced PH.

### Molecular subclassification of human compensated and decompensated right ventricle

**Fig. 6:**
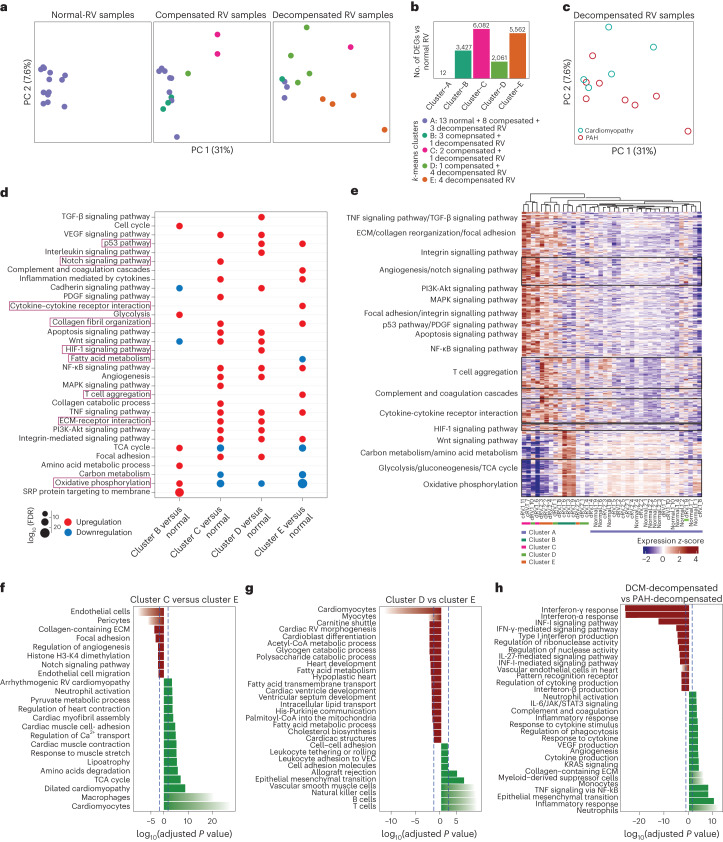
Subclassification of compensated and decompensated states based on the transcriptome in human right ventricle. **a**, PC analysis shows the *k*-means clusters of human RV samples (*n* = 40, *k* = 5). In addition to cluster A, there were two subgroups within compensated samples and two subgroups within decompensated samples. Cluster A contains 13 normal RV, 8 compensated, and 3 decompensated RV samples. Cluster E contains only 4 samples from the decompensated group, while clusters B, D and C each contain a different combination of compensated and decompensated RV samples. **b**, Numbers of DEGs in pairwise comparisons for all clusters versus normal RVs (base mean expression ≥ 5; −0.585 ≤ log_2_FC ≥ 0.585; FDR ≤ 0.05). **c**, Two decompensated RV groups based on their different etiology (PAH or DCM) are shown on the same PCs. **d**, Cumulative enrichment analysis demonstrating the shortlisted important pathways differentially regulated in each cluster (versus control). Size of the dots represents FDR, the red color shows upregulation, while blue represents downregulation of a pathway in the respective pair. Distinguishing pathways are highlighted in red. One-sided Fisher’s exact test was used for all the enrichment analysis, which assesses the independent probability of any genes belonging to any set. FDR-corrected *P* value with Benjamini–Hochberg method was used for multiple-hypothesis testing. **e**, Scaled gene expression (*z*-score) value of 659 genes corresponding to selected dysregulated pathways in human RV clusters of compensated and decompensated samples. Different groups of biological terms with similar regulation are grouped along the *y* axis. **f**–**h**, Pathway analysis of DEGs (*P* adjusted ≤ 0.05; log_2_FC ≥ 1 and ≤ −1) between cluster C (red) and cluster E (green; compensated-2 versus decompensated-2 RV) (**f**), between cluster D (red) and cluster E (green) (decompensated-1 versus decompensated-2 RV) (**g**) and between PAH-associated (green) and DCM-associated (red) decompensated RV subgroups (**h**). The dashed line shows FDR = 0.05. Source data

We further investigated the underlying molecular heterogeneity within the human RV samples by applying a similar unsupervised clustering to the human transcriptome and identified five distinct clusters (A to E; Fig. 6a). To further identify these clusters, we performed DEG analysis for all the clusters compared with the normal RV group (Fig. 6b). Comparison of clusters C and E showed a higher number of DEGs, compared with clusters B and D. Cluster A showed no DEGs as it contains all the normal samples, and a few compensated and decompensated RVs with similar gene expression profiles, which may be caused by their functional similarity, such as in cardiac output, that reflects at the molecular level.

To validate the identity of the clusters, we performed another DEG analysis between the RV subgroups (groups 1 to 6), in which each pair had only compensated or decompensated samples from the same cluster. The results were similar to those of the clusters DEG analysis (Supplementary Fig. 5b and Fig. 6b), which demonstrates a similar identity of clusters with the subgroups of RV. Therefore, we assigned each cluster as a subgroup of compensated (B and C) or decompensated (D and E) RVs, based on the majority of the samples in each. These results suggest that molecular differences between RV subgroups can differentiate the compensated and decompensated RV samples beyond primary hemodynamics classification. Surprisingly, clusters distribution was not different between PAH-associated or dilated cardiomyopathy (DCM)-associated decompensated RV samples (Fig. 6c).

Furthermore, we applied a supervised learning approach (Methods) to see if any of the confounding metadata factors (sex, age, sample type and batches) could predict the specific gene expression pattern observed in the compensated-to-decompensated RV transition. We identified 49 genes with a mean receiver operating characteristic area-under-the-curve (ROC AUC) value ≥ 0.6 among 184 identified bimodal genes (Supplementary Data 3), which revealed that the most impact is driven by sampling batches, while participants’ sex and age did not have significant correlation with the identified genes. However, two other subsets of genes, (independent of sex, age and sample type) were found to be highly expressed in PAH decompensated, and compensated RV, respectively. This suggests that our unsupervised clustering and, therefore, the DEG analysis between the clusters mainly rely on molecular differences within the RV subgroups and are not derived by any of the background factors (Supplementary Fig. 5c).

### Subgroups of human right ventricle show distinct pathway regulation

Accumulative enrichment analysis of each cluster compared to normal RV samples is demonstrated in Fig. 6d, and the heatmap shows the expression of 659 selected genes, corresponding to all the top dysregulated pathways in different RV subgroups (Fig. 6e). Notably, cluster C shows a very similar molecular signature to cluster E, especially in collagen reorganization, inflammatory responses and mitochondrial metabolic shift (Supplementary Fig. 7), which suggests that both clusters have an extremely fibrotic RV, eventually developing end-stage RV failure. This was in line with the participant’s clinical data that confirms severe hypertrophic RV in two of three samples of cluster C. However, in head-to-head differential enrichment analysis, they showed several profound differences such as in notch signaling pathways and citric acid levels (tricarboxylic acid cycle; Fig. 6f).

Moreover, enrichment analysis of DEGs between decompensated subgroups (clusters D and E) revealed impaired fatty acid metabolism in cluster D, while pathways related to EMT, immune response and ECM remodeling were enriched in cluster E (Fig. 6g). Comparing these findings with RV changes in MCT rats, we found one cluster (D) mainly associated with fatty acid dysregulation, similar to early-decompensated RV, while the other (cluster E) showed inflammation, resembling late-stage PAH-associated RV failure. Consequently, the human decompensated subgroups (D and E) were redefined as early-decompensated and late-decompensated phases for further analysis. In addition, enrichment analysis of DEGs between PAH-associated and DCM-associated decompensated RVs, highlighted upregulated inflammation, EMT and distinct ECM regulation in PAH-associated RV failure (Fig. 6h), mirroring cluster E characteristics. Altogether, subgrouping of human RV samples not only unveiled pathways from compensated to decompensated states but also determined diverse RV failure routes in different disease conditions.

### Common regulated pathways in MCT rat and human right ventricular remodeling

**Extended Data Fig. 3:**
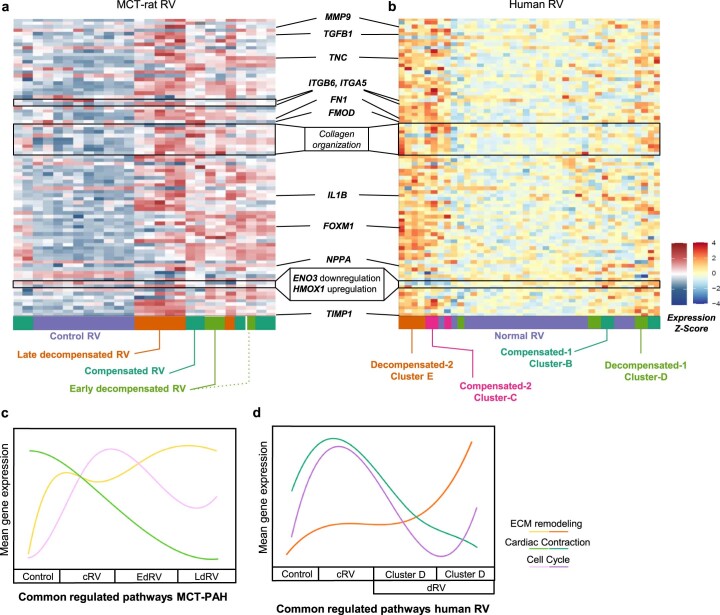
Summary of pathways and genes regulated commonly in human RV dysfunction samples and in MCT-rat RVs. (**a**, **b**) Heatmap for 85 common regulated genes in (**a**) MCT-rat RV and (**b**) human RV datasets. Colors shown on the bottom derived from k-means clustering, representing the samples subgroups of compensated and decompensated RVs. List of gene names are provided in source data. (**c**, **d**) Common regulated pathways in (**c**) MCT-rat and (**d**) human RVs. Human compensated-2 is removed from this representation as it was very similar to decompensated-2. Source data

Combination of MCT rat and human selected DEGs (576 and 659 genes, respectively) revealed 85 commonly dysregulated genes between the two species (Extended Data Fig. 3a,b). Pathway enrichment analysis of these 85 genes highlighted three biological pathways: ECM reorganization, cell cycle and hypoxia regulation (Extended Data Fig. 3c,d). We confirmed expression of several associated genes of each pathway in RV tissues and showed their correlation with RVEDP in MCT rats. Hypoxia-related genes (*HMOX1*, *ENO3* and *ANGPT1*), as well as multiple cell cycle genes (*FGF9*, *WNT5A* and *IL18*) were significantly correlated with RVEDP in MCT rats, suggesting amplification of hypoxic response^23^ and altered glycolytic activity associated with impaired angiogenesis in decompensated RVs^24^ (Supplementary Figs. 8 and 9).

Furthermore, we performed transcription factor (TF) enrichment analysis for the associated DEGs in each subgroup of RVs, in which we identified forkhead box protein M1 (*FOXM1*) protein as a top regulated TF in compensated and early-decompensated RVs of MCT rats, accompanied by other components (E2F TF family, centromere protein A (*CENPA*) and DNA methyltransferase-1 (*DNMT1*)) forming a core regulatory network responsible for cell cycle alterations in the hypertrophic RV in line with previous studies^25^. In addition, FOSL1 turned up as an upstream TF in the late-decompensated RV of MCT rats and its direct association with CEBPE confirms the existence of a pro-apoptotic network activated in the late stage of RV decompensation (Supplementary Fig. 10).

### Extracellular matrix alteration in pulmonary hypertension animal models and human right ventricular remodeling

As mentioned above, ECM remodeling and collagen organization were among the top commonly regulated pathway across species (Fig. 3h). Besides collagens, increased transcription of structural components of cardiac ECM such as fibronectin (FN1) and fibromodulin (FMOD), as well as nonstructural proteins like tenascin (TNC) and thrombospondin (THBS1), was observed in decompensated RV, in line with previous studies of cardiac ECM^26^. In addition, we observed elevated expression of ECM regulators such as matrix metallopeptidase 9 (*MMP9*) and tissue inhibitors of MMPs (*TIMP1*), as well as a significant correlation of *FN1*, *MMP9* and *NPPA* expression with RVEDP (Extended Data Fig. 3 and Supplementary Fig. 11), which corroborates evidence of active collagen remodeling and the fibrotic phenotype of end-stage RV failure. Among all the differentially expressed ECM genes, considering the DEGs between PAH-associated versus DCM-decompensated RVs, and late-versus-early-decompensated subgroups, we shortlisted 13 ECM-related target genes, for further validation at the protein level in PAH.

**Extended Data Fig. 4:**
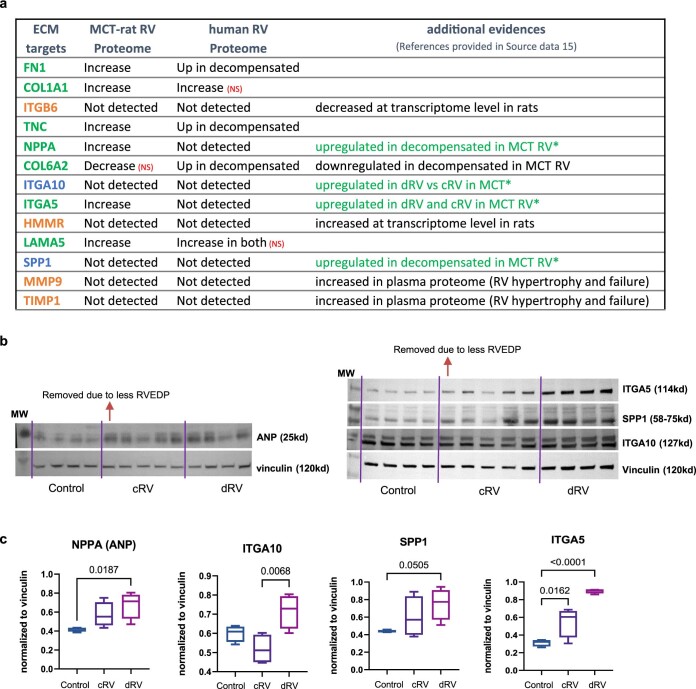
Public proteome and Western blot validation of selected ECM proteins in MCT-RV and human RV. (**a**) Summary of 13 non-biomarker targets associated with ECM from the transcriptome analysis, along with their evidence of protein expression levels in animal models and human RV from publicly available datasets. The western blot analysis results has been highlighted in green in the third column. (**b**) Western blot images for ANP, ITGA5, ITGA10, and SPP1, in control (n = 4), cRV (n = 5) and dRV (n = 4) samples from MCT-induced rats. ANP is loaded in a separate blot from three other proteins. (**c**) Western blot quantifications for ANP, ITGA5, ITGA10, and SPP1 normalized to the corresponding loading control of each blot (Vinculin). Protein expression levels has been tested by one-way ANOVA followed by Dunnett’s multiple comparisons test (exact P-value has been demonstrated when significant). Data are presented as mean ± SEM. In all the box plots: Central bands represents 50% quantile (median), box interquartile ranges: 25–75%, and whiskers set to max/min, 1.5 IQR above/below the box. Source data

Next, to confirm the alteration of those 13 ECM-associated transcripts at the protein level, we evaluated publicly available proteome datasets from human RV samples as well as different animal models. We primarily found 7 proteins to be regulated in 4 RV proteomics from different PH models as well as PAH participants^7–11^ (Extended Data Fig. 4a). We confirmed the remaining ECM molecules (ITGA10, ITGB6, HMMR, MMP9, SPP1 and TIMP1) with no proteomics record in public datasets, as either regulated in plasma of PAH participants (MMP9 and TIMP1)^12,14^, or similarly regulated in transcriptomes from other rat models of PH^5,6^. The remaining molecules were confirmed by western blotting in MCT rat RV tissues (Extended Data Fig. 4b,c).

### Integration of right ventricular transcriptomes with pulmonary arterial hypertension plasma proteomes

**Fig. 7:**
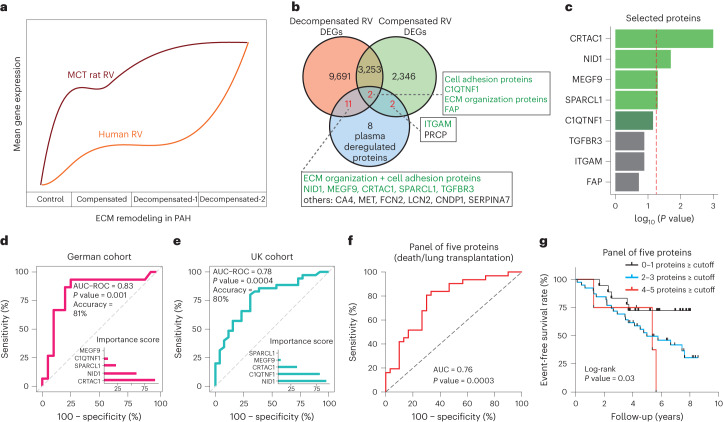
Plasma levels of five extracellular matrix regulatory proteins act as a potential prognostic biomarker for pulmonary arterial hypertension. **a**, Transcriptome regulation of ECM-associated genes in both human and MCT rat RV samples. **b**, Venn diagram showing the inclusion of all dysregulated genes (base mean expression ≥ 5; −0.585 ≤ log_2_FC ≥ 0.585; FDR ≤ 0.05) from human RV transcriptomes, and top 25% of deregulated plasma proteins between participants with compensated and decompensated RV in the discovery PAH cohort. Proteins associated with ECM or cell adhesion are highlighted in green. **c**, Common ECM regulatory proteins between transcriptomes and the PAH proteome cohort, and their corresponding *P* value was calculated by unpaired *t*-test comparison between compensated and decompensated subgroups of participants in the discovery cohort (CRTAC1 = 0.001, NID1 = 0.02, MEGF9 = 0.05, SPARCL1 = 0.05, C1QTNF1 = 0.07, TGFBR3 = 0.13, ITGAM = 0.13, FAP = 0.19). *P* values were not corrected for multiple comparisons. Top five proteins selected to check for their biomarker capacity are highlighted in green. The dashed line highlights *P* value = 0.05. **d**,**e**, ROC analysis and the corresponding AUC and accuracy showing the performance of the random forest model for the panel of five proteins in classifying two groups of participants (decompensated versus compensated) in the discovery cohort (German) (**d**) and validation cohort (UK) (**e**). ROC *P* value was calculated using one-sided Mann–Whitney (Wilcoxon-based) test for the H0: AUC = 0.5, and not corrected for multiple comparisons. Feature (proteins) importance score measurement in each cohort, showing the relative influence of each protein in prediction performance. **f**, ROC curve measuring the risk of death/lung transplantation in participants from the second PAH cohort during years of follow-up counting for a combination of five proposed biomarkers to assess the optimal cutoff value and their performance in predicting transplant event-free survival. The ROC accuracy was tested with the one-sided Wilson/Brown method for 95% confidence intervals, and the *P* value was not corrected for multiple comparisons. **g**, Kaplan–Meier analysis shows the transplant event-free survival rate and a log-rank test *P* value for the comparisons between groups using a panel of five proteins based on optimal cutoff levels from the ROC curves of each protein. Participants were divided into three groups based on the total number of proteins that had cutoff levels equal to or greater than the optimal level. Black line, 0–1(proteins); blue line = 2–3 (proteins); red line, 4–5 (proteins).

**Extended Data Fig. 5:**
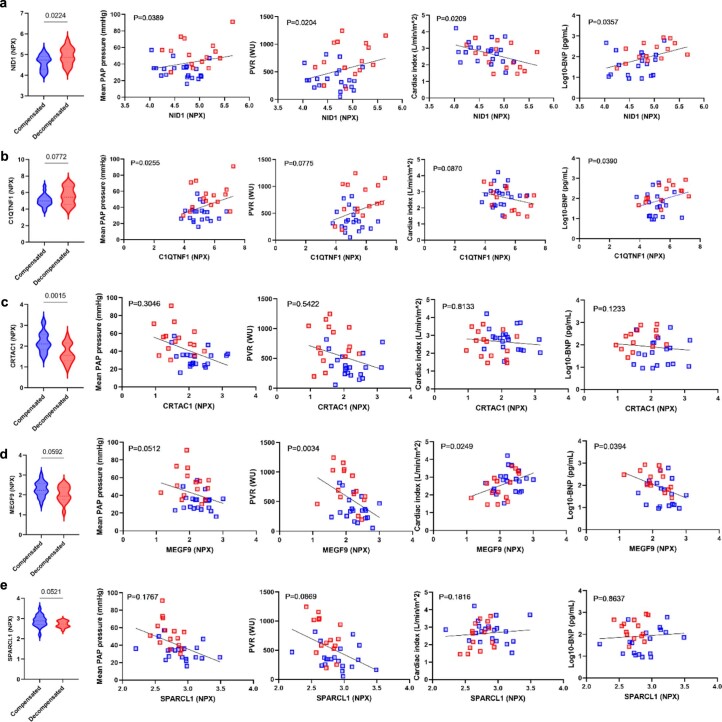
Expression and functional correlation of selected biomarker candidates in German cohort. Protein expression levels (NPX), and simple linear regression for each protein with RV functional parameters in two groups of PAH patients (compensated and decompensated). (**a**, **b**) NID1 and C1QTNF1 were two proteins with absolute circulating upregulation from compensated to decompensated RV conditions, while correlating significantly with all the all the functional parameters for NID1, and with mPAP and proBNP, for C1QTNF1 (**c**, **d**). CRTAC1 and MEGF9 show downregulation in decompensated vs compensated. MEGF9 correlates highly with PVR, CI, and proBNP levels (**d**), while CRTAC1 does not have significant correlation with any of the parameters (**c**). SPARCL1 shows slight downregulation in decompensated vs. compensated RV, with no significant functional correlation (**e**). Protein expression levels has been tested by two-tailed unpaired t-test, and *p-value* has not been corrected for multiple comparison as it does not apply. Data are presented as mean ± SEM. multiple linear regression has been applied for each protein vs RV functional parameter, while the variance analysis *P-value* has been adjusted for age and sex. (mPAP = mean pulmonary arterial pressure, PVR= pulmonary vascular resistance, CI = Cardiac Index). Source data

To evaluate the potential of dysregulated ECM proteins (the predominantly dysregulated pathway in both human and rat RV transcriptomes; Fig. 7a) as circulating biomarkers for PAH-associated RV dysfunction, we performed a proteomic profiling from the plasma of 35 PAH participants including 20 participants with compensated and 15 with decompensated RV states, serving as discovery cohort (German). Classification of compensated and decompensated RV was done based on the RV end-systolic elastance (Ees)/arterial elastance (Ea) ratio measured by magnetic resonance imaging (MRI) as previously described^19^, and Ees/Ea = 0.8 was considered as the cutoff (Methods). Using an unbiased approach, we first selected the top significant DEGs in all pairs of our transcriptomes (*P* < 0.05), which included about 30% of total transcripts (15,305 genes). Then, we selected the top 25% of differentially expressed proteins of our discovery cohort (23 proteins). Next, we combined both differentially regulated candidates from transcriptomes and plasma proteomes to find common targets. Interestingly, more than 60% of plasma regulated proteins (15 of 23) were commonly dysregulated in both datasets, where 8 proteins were functionally linked to ECM remodeling or cell adhesion (Fig. 7b). We selected those 8 ECM proteins for downstream analysis, and cross-checked their differentially regulation in plasma between compensated and decompensated groups in the corresponding proteome cohort. Of the 8 proteins, 5 (CRTAC1, NID1, SPARCL1, MEGF9 and C1QTNF1) were selected to continue further confirmation analysis (Fig. 7c and Methods). Among these 5 proteins, NID1 and C1QTNF1 were upregulated in participants with decompensated RVs, whereas the other 3 were upregulated in the participants with compensated RV (Extended Data Fig. 5).

### Plasma levels of extracellular matrix proteins act as potential biomarkers

We then assessed the clinical correlation of these five deregulated ECM proteins with mean pulmonary arterial pressure (mPAP), pulmonary vascular resistance (PVR) and cardiac index (CI), as well as N-terminal prohormone of brain natriuretic peptide (NT-proBNP levels) in the PAH participants. Increased protein expression of NID1 and C1QTNF1 was significantly correlated with increased mPAP and proBNP levels, while NID1 was also significantly correlated with increased PVR and decreased CI. In contrast, a decreased level of MEGF9 was strongly correlated with PVR, CI and proBNP levels, altogether suggesting a strong association of ECM protein regulation with RV-associated prognostic factors of PAH in our discovery cohort (Extended Data Fig. 5).

**Extended Data Fig. 6:**
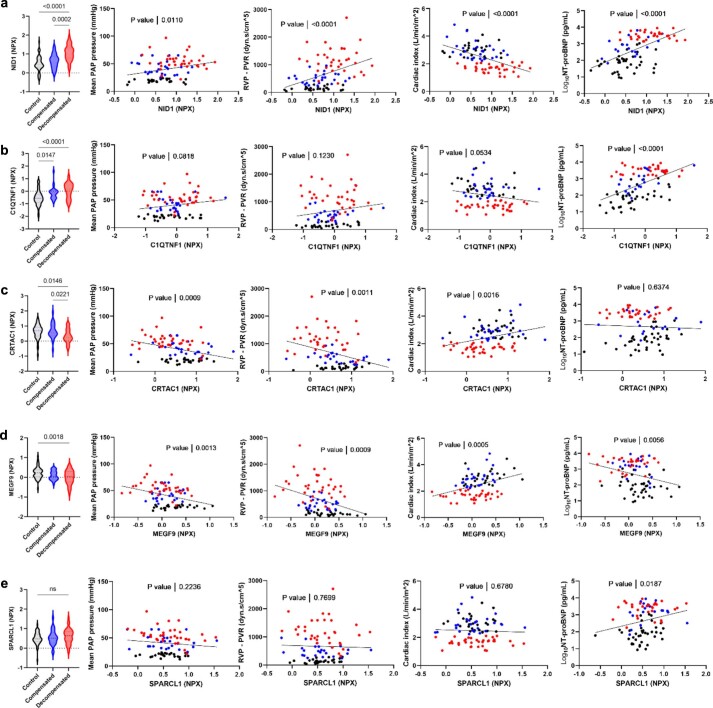
Expression and functional correlation of selected biomarker candidates in UK cohort. Protein expression levels (NPX), and simple linear regression for each protein with RV functional parameters in two groups of PAH patients as well as controls. (**a**) NID1 expression was significantly correlated with all the hemodynamics parameters, negatively with Cardiac Index, while positively with mPAP and PVR as well as proBNP levels. (**b**) C1QTNF1 expression was positively correlated only with proBNP levels. (**c**, **d**) CRTAC1 and MEGF9 were both significantly correlated with all the clinical parameters, negatively with mPAP and PVR, and positively with Cardiac Index. (**e**) SPARCL1 did not show any significant correlation with measured clinical parameters except for a positive correlation with proBNP levels. Protein expression levels has been tested by one-way ANOVA followed by Turkey’s multiple comparison test. Data are presented as mean ± SEM. Simple linear regression has been applied to calculate the *P-value* of protein expression corrlation vs RV functional parameters. (mPAP = mean pulmonary arterial pressure, PVR = pulmonary vascular resistance, CI = Cardiac Index). Source data

To validate these results, we analyzed expression levels of all five proteins in an independent plasma proteomics analysis of a PAH cohort (United Kingdom) including 61 PAH participants and 56 controls (Methods). Notably, we observed a significant increase in the expression of NID1 and C1QTNF1 as well as decreased expression of CRTAC1 and MEGF9 in participants with decompensated RV compared to participants with compensated RV (Extended Data Fig. 6) in line with the discovery cohort findings. Similarly, NID1 showed a negative correlation with RV functional parameters such as CI, while a positive correlation with mPAP and PVR, and proBNP levels, was observed; however, this effect was opposite for downregulated proteins (CRTAC1 and MEGF9). C1QTNF1 and SPARCL1 showed a positive correlation only with proBNP levels in the validation cohort (Extended Data Fig. 6).

**Extended Data Fig. 7:**
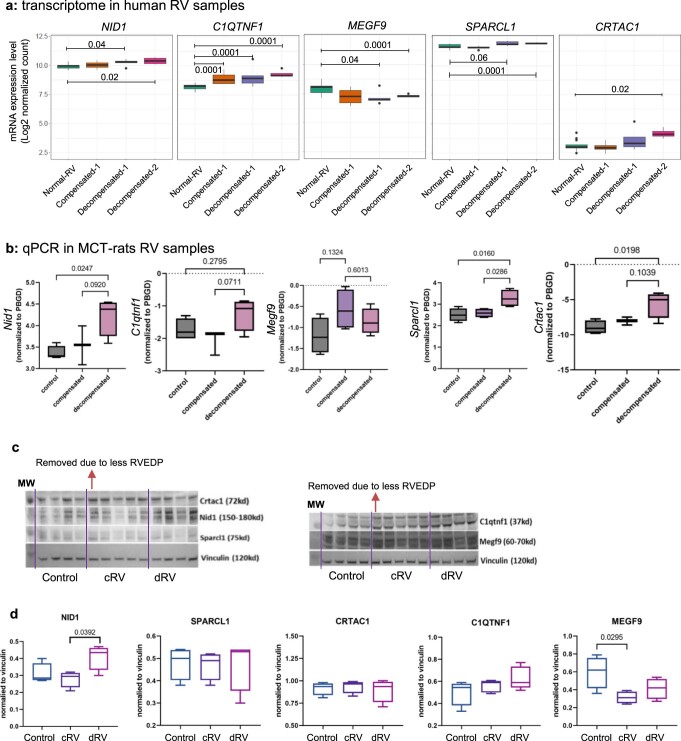
Transcriptome, qPCR and Western blot validation of selected biomarkers in human and MCT-induced rat RV. (**a**) Transcriptome level of five biomarker genes associated with ECM in normal (n = 13), compensated (n = 4) and decompensated-1 (n = 5), and decompensated-2 (n = 4) subgroups of RV. Central band: 50% quantile, box: interquartile range (25–75%); whiskers: max/min are 1.5 IQR above/below the box. (**b**) Relative mRNA expressions measured by qPCR for 5 selected biomarkers in control (n = 5), compensated (n = 5) and decompensated RVs (n = 5) from MCT-induced rats. (**c**) Western blot images for NID1, SPARCL1, CRTAC1, C1QTNF1, and MEGF9 in control (n = 4), cRV (n = 5) and dRV (n = 4) samples from MCT-induced rats. First three proteins are loaded in a separate blot from two others. (**d**) Western blot quantifications for 5 selected proteins, normalized to the corresponding loading control of each blot (Vinculin). Both mRNA and protein expression levels has been tested by one-way ANOVA followed by Tukey’s multiple comparisons test. Exact *P-value* has been demonstrated where significant. Data are presented as mean ± SEM. In all the box plots: Central bands represents 50% quantile (median), box interquartile ranges: 25–75%, and whiskers set to max/min, 1.5 IQR above/below the box. Source data

To determine whether plasma levels correspond to changes in RV tissues, additional quantitative PCR with reverse transcription (RT–qPCR) and western blot analyses of these five molecules in MCT-induced rats and the comparison with their human transcriptome were performed. Upregulation of NID1 and C1QTNF1 and downregulation of MEGF9 at both RNA and protein levels in RV tissue corresponded with their plasma level alterations in PAH participants. However, the observed downregulation pattern of two other proteins (especially CRTAC1) in the plasma contrasted with the increased mRNA levels in the RV, which could be due to the lower protein expressions in RV, or the secretion is more pronounced in other PAH-affected organs such as the lung (Extended Data Fig. 7)^27,28^.

### Plasma signature of five extracellular matrix proteins classifies right ventricular states

**Extended Data Fig. 8:**
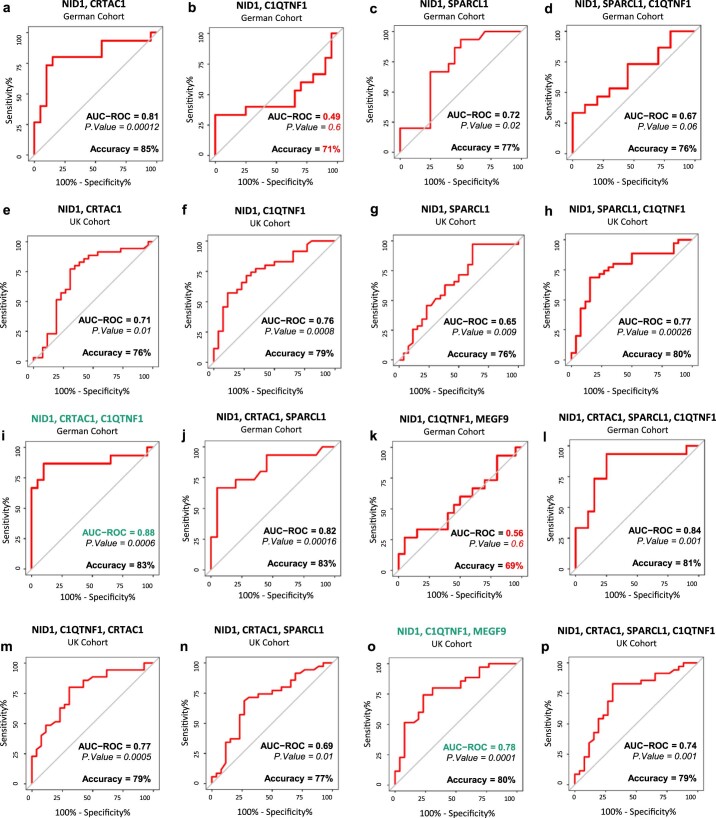
Combination of five proposed biomarkers can predict compensated vs decompensated in both cohorts. ROC curves (associated AUCs and accuracy) indicating Random forest model performance in classifying patients with compensated or decompensated RV in each cohort independently. *P-value* has been calculated using one-sided Mann-Whitney (Wilcoxon-based) test for the H0: AUC = 0.5, and not corrected for multiple comparison as not applicable here. (**a–p**) Different combinations of proteins from panel of five has been tested to find the most effective features in prediction of RV states. Combination of NID1 + C1QTNF1 + CRTAC1 in German cohort (**i**) and NID1 + C1QTNF1 + MEG9 in validation cohort (**o**) are the most significant classifying features (highlighted in green).

We further assessed the ability of these five proteins to predict RV dysfunction and disease severity in PAH using a random forest model (Methods), followed by a ROC curve analysis. We demonstrated that the panel of five proteins could significantly differentiate between compensated and decompensated groups of PAH participants in our discovery cohort with ROC AUC = 0.83 (*P* = 0.001, accuracy = 81%; Fig. 7d). Based on the calculated feature importance score (Methods), CRTAC1 and NID1 were highly predictive in the discovery cohort (Fig. 7d), while in the validation cohort, NID1 and C1QTNF1 had the most predictive performance in discriminating compensated and decompensated states of the RV samples of PAH participants (Fig. 7e). Considering both cohorts, this result suggests NID1 demonstrates the most significant biomarker potential, while the combination of NID1 with C1QTNF1 and CRTAC1 had highest overall performance compared to other combinations in discriminating two subgroups of PAH (AUC = 0.88 and 0.77 in discovery and validation cohorts, respectively; Extended Data Fig. 8).

**Extended Data Fig. 9:**
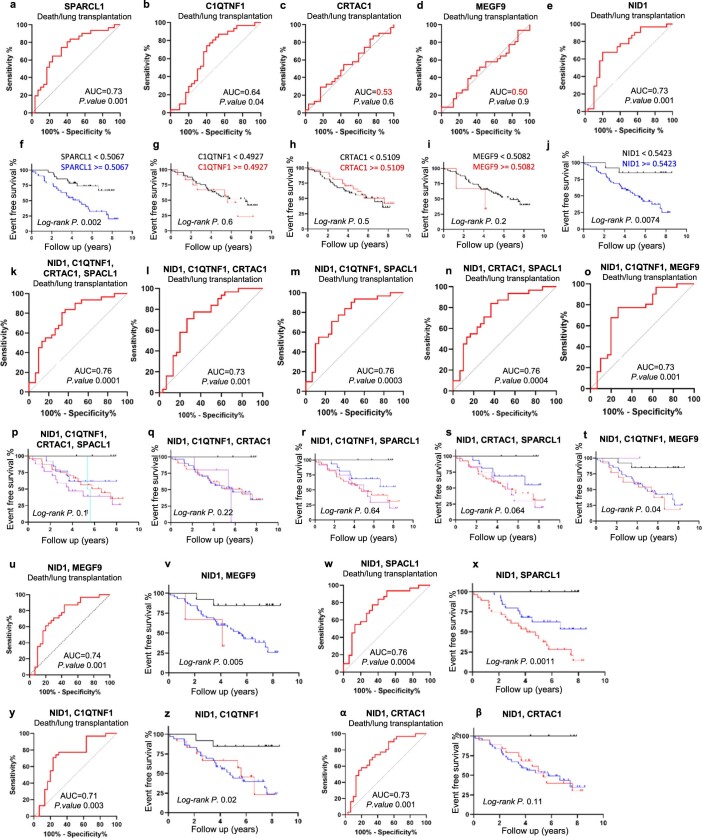
Different combinations of five proposed biomarkers can predict death/lung transplantation in PAH patients. (**a**–**e**, **k–o**, **u**, **w**, **y**, **α**) ROC curve for the risk of death/lung transplantation in patients (UK cohort) during years of follow-up counting for each single or different combination of 5-proteins panel, to assess the optimal cut-off value and their performance in predicting event-free survival (**f**–**j**, **p**–**t**, **v**, **x**, **z**, **β**). The ROC accuracy has been tested with one-sided Wilson/Brown method for 95%CI, and *P-value* has not been corrected for multiple comparison as not applicable here. The Kaplan–Meier analysis shows the event-free survival rate and a log-rank test p-value for the comparisons between groups for each single or combination of proteins (survived vs death/transplantation) using the optimal cut-off from the ROC curves. The numbers of proteins that had equal or more expression than cut-off level was summed and patients divided into different groups based on the the summed values, (**f**, **j**) to two groups: black= SPARCL1/NID1 expression lower than cutoff and, blue = SPARCL1/NID1 expression higher than cutoff. (**g**–**i**) two groups: Black = C1QTNF1/CRTAC1/MEGF9 lower than cutoff, red = C1QTNF1/CRTAC1/MEGF9 expression higher than cutoff. (**v**, x, z, ß) to three groups: black = both expression lower than cutoff, blue= one cutoff criteria met, red = both expression higher than cutoff. (**q**–**t**) Patients were divided by the number proteins that have equal or more than cut-off level to four groups: Black = 0 Blue = 1 Red = 2 Purple = 3 proteins. (**p**) Patients were divided by the number proteins that have equal or more than cut-off level to five groups: Black = 0 Blue = 1 Red = 2 Purple= 3 Cyan = 4 proteins. NID1 + SPARCL1 have the most ROC-AUC, while NID1 + MEGF9 (also in combination with C1QTNF1/CRTAC1) shows a high AUC for death/lung transplant vs survival prediction. Event-free survival is better correlated with NID1, SPARCL1, and MEGF9.

To evaluate the potential of these five proteins to predict survival, we used participant follow-up data from the validation cohort. Among 61 PAH participants, 31 were deceased or received lung transplantation, while the other 30 survived during the median follow-up of 4.5 years. Interestingly, ROC analysis revealed that the panel of five proteins could significantly discriminate PAH participants with lower survival outcome (Fig. 7f,g). NID1 and SPARCL1 stand as independent predictors for PAH participants’ survival (AUC = 0.73 for both; Extended Data Fig. 9a–j), while the combination of NID1 with SPARCL1 or MEGF9 increased the survival prediction performance to AUC = 0.76 and 0.74, respectively (Extended Data Fig. 9).

Furthermore, transplant-free survival estimation was performed based on the cutoff values introduced by ROC analysis of single proteins and for all the possible combinations (Methods and Extended Data Fig. 9), in which we confirmed NID1 and SPARCL1 can discriminate PAH participants with poorer transplant-free survival (log-rank *P* value = 0.007 and 0.002, respectively), while NID1 in combination with each of the other proteins (SPARCL1, C1QTNF1 or MEGF9) remained significantly predictive.

### NID1 and C1QTNF1 distinguish early- and late-decompensated right ventricle

**Fig. 8:**
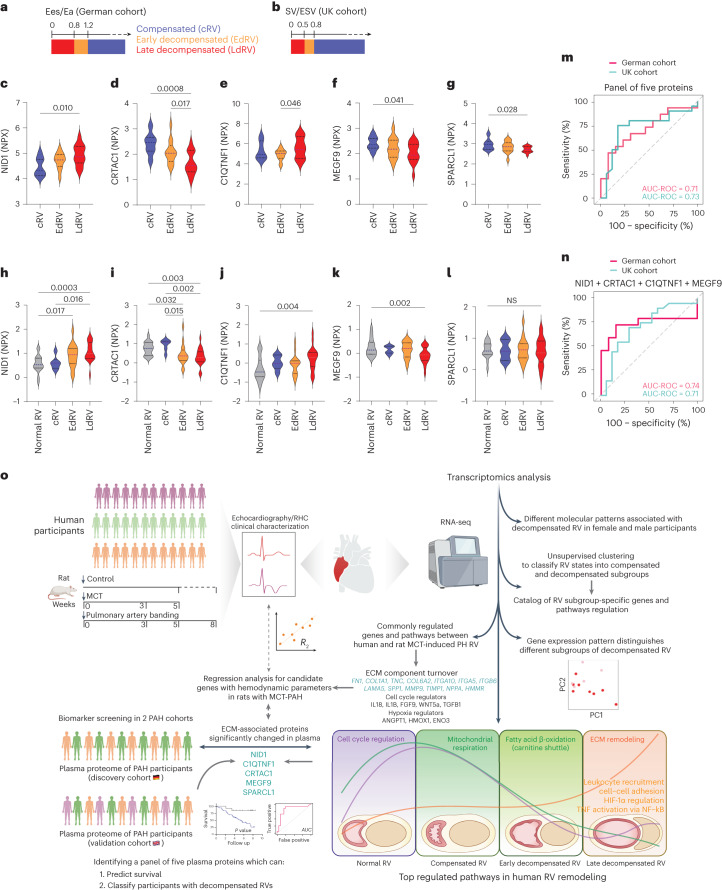
NID1 and C1QTNF1 proteins can distinguish early- to late-decompensated right ventricle in pulmonary arterial hypertension. **a**–**b**, Representation of cutoff that was used for definition of early and late decompensation based on Ees/Ea ratio in the discovery cohort (**a**) and based on SV/ESV ratio in the validation cohort (**b**). **c**–**g**, Plasma levels of five selected proteins in the discovery cohort (German). **h**–**l**, Plasma levels of five selected proteins in the validation cohort (UK). *P* values were calculated by one-way ANOVA. **m**,**n**, ROC curve for both cohorts showing the prediction performance for the panel of five proteins (discovery *P* value = 0.07, validation *P* value = 0.06) (**m**) and for only four proteins, excluding SPARCL1 (discovery *P* value = 0.08, validation *P* value = 0.08) (**n**), in classifying participants with late- versus early-decompensated RV. EdRV, early-decompensated right ventricle; LdRV, late-decompensated right ventricle. **o**, Schematic summary of the study design and main results. Human participants and rat models were subjected to echocardiography and RHC, and RV samples were obtained for RNA profiling (top left). Workflow for the transcriptomic analysis of the RV samples indicating the subgrouping approach (top right). Identification and validation of five plasma proteins, associated with ECM which can predict survival and classify participants with different RV conditions (bottom left). Summary of the most dysregulated pathways in human RV remodeling during PAH. The RV samples were divided into four distinct phenotypes: normal, compensated, early decompensated and late decompensated, based on integrative findings in RVs from both human and MCT-PH rats (bottom right). Created with BioRender.com.

**Extended Data Fig. 10:**
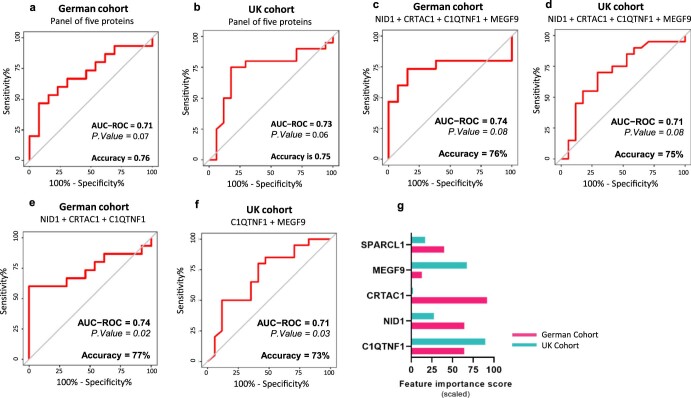
Classification of PAH decompensated RVs to early and late using panel of five potential biomarkers. Extended ROC curves (associated AUCs, accuracy, and *P-values*), using panel of five proteins; (**a**, **b**) for prediction of late vs. early decompensated in both cohorts, as well as four proteins excluding SPARCL1 (**c**, **d**), and the most effective factors of each cohort independently (**e**, **f**). ROC-AUC values indicate the random forest model performance for discriminating patients with early or late decompensated RV. *P-value* has been calculated using one-sided Mann-Whitney (Wilcoxon-based) test for the H0: AUC = 0.5, and not corrected for multiple comparison as not applicable here. (**g**) Relative feature importance score for the performance of each protein in prediction of late vs. early decompensated RV in patients in both cohorts (related to Fig. 8m).

Moreover, we investigated whether the panel of five proteins can predict the early stage versus the late stage of RV dysfunction. In this regard, we utilized participant MRI data to characterize early and late stages of RV decompensation in both cohorts. Because RV–PA coupling mirrors right ventricular adaptation and provides a very sensitive measure of early adaptation and maladaptation, we decided to define the different stages of RV decompensation based on the ratio of end-systolic elastance to afterload. In our discovery cohort, we used a gold-standard pressure volume loop analysis to define Ees/Ea as described before^19^, while in the validation cohort, the ratio of SV to end-systolic volume (SV/ESV), a validated surrogate was used, based on MRI data as previously established^29,30^. Thus, participants with 0.8 < Ees/Ea < 1.2 or 0.5 < SV/ESV < 0.8 were considered as having early-decompensated RV, and participants with Ees/Ea < 0.8 or SV/ESV < 0.5 were considered as being in the late-decompensated state (Fig. 8a,b). We then compared the plasma expression levels of five proteins in the identified decompensated subgroups as well as in participants with compensated RV. Interestingly, C1QTNF1 and CRTAC1 showed a significant regulation between early- to late-decompensated RVs, while NID1, MEGF9 and SPARCL1 were strongly regulated only in the late stage of decompensation in discovery cohort (Fig. 8c–g). In the validation cohort, NID1 and CRTAC1 showed a significant dysregulation in PAH patients with early-decompensated RV and further in the late stage, while C1QTNF1 and MEGF9 were regulated only in late-stage PAH patients with decompensated RV (Fig. 8h–l). Irrespective of slight differences in the regulation between the two cohorts, the panel of five proteins enabled us to discriminate late-versus-early decompensation RV states of PAH participants (AUC ROC = 0.71 and 0.73 in both cohorts, respectively; Fig. 8m). Moreover, four ECM proteins were enough to significantly predict early versus late stages of RV decompensation in PAH participants, which is in line with less significant changes in SPARCL1 protein levels both in plasma and RV (Fig. 8n). Furthermore, feature importance measurements indicated that NID1 and C1QTNF1 have the highest overall performance in both cohorts in predicting the late stage of RV decompensation, along with CRTAC1 or MEGF9 as an additional promising factor in both cohorts, respectively (Extended Data Fig. 10).

## Discussion

In this study, utilizing human RV and rodent models of RV dysfunction (MCT and PAB), we determined a comprehensive transcriptomic signature of RV hypertrophy (compensated) and failure (decompensated). By integrating plasma proteomics of two independent PAH participants cohorts, we further revealed several important findings (Fig. 8o). First, unsupervised clustering revealed the presence of molecular subgroups within each compensated and decompensated RV states beyond their hemodynamics characterization. Second, we identified and validated several molecular pathways associated with ECM, cell cycle, energy metabolism and hypoxia, TNF and other inflammatory factors along with their key corresponding genes that underlie the pathological transition from adaptive to maladaptive RV to failure. Third, the sex-specific differences in RV adaptation in both human and MCT rats have been deciphered. Fourth, we provided evidence for five potential ECM-related biomarkers (NID1, C1QTNF1, CRTAC1, MEGF9 and SPARCL1) that were altered at both RV transcriptome and plasma proteome levels, which could classify compensated and decompensated RV and predict worse clinical outcomes in PAH participants. Lastly, we showed plasma levels of NID1 and C1QTNF1 can determine early- versus late-decompensated RV states in PAH participants. Integrative transcriptomic analysis of RV remodeling with plasma proteins suggests a path toward the identification of state-specific biomarkers and therapeutic targets using the omics-based phenotyping of RV in PAH.

This study aimed to compare compensated and decompensated RV from human and two well-known PH animal models (MCT and PAB-induced RV dysfunction). ECM remodeling emerged as the most dysregulated pathway across all datasets, in addition to similar inflammatory responses in the decompensated RV across all datasets, while molecular signatures of MCT rat decompensated RV were stronger than in PAB and, therefore, resembled human RV dysfunction better. However, each animal model has unique features and drawbacks. MCT closely mirrors human RV remodeling, but itself affects the heart^31^. In contrast, the PAB model focuses on vascular-independent mechanisms, potentially preventing severe RV failure. Our comprehensive RV transcriptomic profiles stand out, confirming a continuous transition through initial adaptive stages, to the uncoupled maladaptive stages^32^, and uniquely identifies genes and pathways linked to each RV stage/subgroup, providing deeper insights into disease mechanisms.

We identified two compensated and two decompensated states of human RV based on molecular signature. A subgroup of human compensated RV (cluster C) with a similar signature to end-stage decompensated RV (cluster E) suggested that a particular compensatory RV state could develop (faster) into maladaptive hypertrophy. Furthermore, comparison of two decompensated RV subgroups showed that strong reorganization of ECM starts in early stages, while it also majorly impacts on fatty acid dysregulation. Moreover, specific pathways such as TNF activation via NF-kB, EMT and activated inflammatory response could discriminate PAH-associated decompensated RV versus the non-PAH-decompensated RV.

RNA-seq analysis of RV samples from human and MCT rats, including both sexes, provided valuable insights into the sex-specific RV adaptation. The results highlighted that despite key changes in the transition from the compensated to decompensated RV independent of sex, females may develop a different route to decompensated RV and prolong the compensated phase. We observed that the protective mechanism against decompensated RV in female animal models mainly depends on fatty acid metabolism, whereas the deterioration signature with massive remodeling of the ECM occurs in the earlier phase in males, in line with previously published findings^33^. Similarly, cell death signaling is activated earlier in male PAH participants, whereas the decompensated phase in females is strongly regulated by hypoxic metabolism, estrogen response and fatty acid regulation. Notably, differential TF complexes (HIF-1A/NF-kB1 versus IRF5/EGR2), as well as distinctive histone demethylase activity (Kdm6a versus Kdm5d) showed that sex differences observed during RV remodeling are caused not only by sex hormones, but also by sex chromosome-mediated epigenetic mechanisms^34^.

Moreover, ECM component alteration was predominant in our study. As described before^35^, myocardial fibrosis, mainly due to the reorganization of collagen fibrils, plays a crucial role in the development of RV failure^1,35^, and in the formation of both adaptive and maladaptive RV responses in PH^36,37^. We demonstrated that increased transcription of ECM components occurs in the decompensated RVs of both PH animal models and PAH participants. Validation of selected ECM molecules at the protein level^7–11^, along with western blotting in RV tissues suggested a high correlation between mRNA and protein abundance, indicating their potential role in ECM remodeling that facilitates the transition from adaptive to maladaptive RV in PAH.

Combined analysis of RV transcriptome and plasma proteomes from PAH participants confirmed deregulation of five ECM molecules that serve as cardiac-specific biomarkers. Among these, C1QTNF1 (refs. ^38–42^), SPARCL1 (refs. ^43,44^) and CRTAC1 (ref. ^45^) have been previously shown as biomarkers for various cardiovascular diseases. In addition, the roles of NID1 (ref. ^46^), C1QTNF1 (refs. ^42,47^), SPARCL1 (ref. ^48^), CRTAC1 (ref. ^45^) and MEGF9 (ref. ^49^) in cardiac remodeling confirmed their relevance to and the prognostic significance for RV dysfunction in PAH. Notably, the panel of five proteins (also when excluding SPARCL1) could discriminate compensated versus early and late stages of RV decompensation in both cohorts. NID1 (also in combination with the other four) could significantly discriminate PAH participants with lower survival, which suggests the panel of five ECM proteins can serve as a prognostic biomarker alone or in combination with other diagnostic methods.

The major limitation in molecular profiling (omics) approaches, as in our study, is the limited access to clinical samples from humans at different stages of RV disease, as well as lacking the longitudinal assessment during disease progression, resulting in a low number of samples for comprehensive computational analysis. In the same regard, we must be cautious in drawing the indisputable conclusion, as the low number of samples are likely to be associated with insufficient performance power in machine learning approaches such as clustering and linear regression (resulting in lower ROC). Secondly, the heterogeneity within human RV samples, which reflects the etiologic complexity underlying the pathophysiology of RV dysfunction in PAH, mitigates the differential expression discoveries, and therefore transcriptomic data may reflect dysregulation of regulatory mechanisms associated with disease progression partially. Nevertheless, transcriptome profiling is still a valuable technique for mapping the structural and functional alterations of RV myocardium.

Additionally, despite high concordance between mRNA and protein abundance in RV tissues, a proteomic study provides more valuable insights. On the other hand, the low number of gene–protein matches when integrating proteomic with transcriptomic datasets may be explained by posttranslational regulations, but also by the low coverage and sensitivity of current proteomic assays, especially regarding ECM components of cardiac proteomics^50^. Similarly, the lower detection rate of the O-link panel used for biomarker screening may limit the discovery aspect^51^. Future efforts in the technological development of clinical proteomics, focusing on increasing the specificity and quantity of proteins, as well as cell-specific discovery approaches, are essential to unravel the stage-specific regulatory landscape of RV dysfunction associated with cardiopulmonary disease.

## Methods

### Inclusion and ethics

Studies with human RV tissues or cells were performed with the approval of Laval University and the Biosafety and Ethics committees of the University Institute of Cardiology and Respirology of Quebec (CER 20773, CER 20735, CER 21747). Preclinical PAH experiments in rats were performed according to the guidelines of the Canadian Council on Animal Care and approved by the Animal Care and Use committees of Laval University (2014-176 and 2018-015). Studies on PAH plasma were performed with the approval of the local ethics committee (AZ 58/15) at University Hospital Giessen and Marburg, Department of Pneumology and Critical Care Medicine, Germany from 2016 to 2018 for the German cohort. Participants in this study signed an informed consent form before the sample collection. The UK cohort plasma proteome analysis was done with the approval of Laval University and the IUCPQ Biosafety and Ethics Committees (CER 20735), and the University of Sheffield from the Sheffield Teaching Hospitals Observational Study of Patients with Pulmonary Hypertension, Cardiovascular and Lung Disease (UK REC ref. 18/YH/0441) from 2013 to 2018. All participants gave informed consent to be part of the study beforehand.

### Human right ventricular tissue collection

Our experimental procedures for using human tissues or cells conformed to the principles outlined in the Declaration of Helsinki. They were performed with the approval of Laval University and the Biosafety and Ethics committees of the University Institute of Cardiology and Respirology of Quebec (CER 20773, CER 20735, CER 21747). All experiments were performed in accordance with the latest preclinical PAH research guidelines^52,53^. Tissues were obtained from participants who had previously given written, informed consent. Initially, 45 participants (male and female) were classified as control, or with compensated, or decompensated RV condition, based on clinical history and CI. The procedures and criteria for the acquisition of control, compensated and decompensated RV samples were as previously described^20^. In brief, participants with RV dilation, preserved CI (>2.2 l min^−1^ per m^2^), or normal (≥17 mm) tricuspid annular plane systolic excursion measured by echocardiography were considered as compensated hypertrophy, whereas PAH participants who received a lung transplantation or who died with RV failure were considered as those with decompensated RV. Normal distribution of sex per group of participants (normal/PAH and compensated/decompensated) was tested by Fisher’s exact test to confirm that there was no significant effects of sex in any of the comparisons (Fig. 4a).

### Animal model of pulmonary hypertension and right ventricular hypertrophy

Experiments were performed according to the guidelines of the Canadian Council on Animal Care and approved by the Animal Care and Use committees of Laval University (2014-176 and 2018-015). For the animal studies, male and female Sprague Dawley rats (*Rattus norvegicus*) at age 8–12 weeks, (Charles River Laboratories) were subjected to the treatment protocol as previously described^20^. In brief, for the MCT model, a single subcutaneous injection of MCT (60 mg per kg body weight) was applied (control rats received saline), and the RV function was monitored weekly using echocardiography. Terminal right heart catheterization (RHC) was performed on anesthetized, closed-chest rats. The whole experiment procedure was 5 weeks for the MCT rat model.

For the PAB model, following anesthesia, the PA was separated from the aorta and left atrium, and was tied against a 19-gauge needle and then released quickly. PAB-operated rats with peak velocities > 3.5 m s^−1^ per m^2^ at the banding site (assessed by echocardiography), as well as sham-operated rats without tying the pulmonary trunk were included for the study. Between weeks 3 and 8 following PAB operation, sham and PAB-operated rats were euthanized following echocardiography, and underwent terminal RHC at the end of protocol, based on clinical symptoms of RV failure. The numbers of initial animal groups were 30, 30 and 15 for first and second batches of MCT and PAB experiments, respectively. The number of samples left after removing the outliers were 26, 27 and 14, respectively.

We classified rat RVs into control, compensated RV and decompensated RV (and into early or late decompensated for the MCT model) as previously described^20^, according to CO, RVEDP and clinical RV failure signs. After performing measurements, the heart, lungs and other major organs were harvested.

### Histological analyses

RV fibrosis and cardiomyocyte cross-sectional area analysis was performed as described before. Briefly, rat RVs were harvested, fixed with 4% formaldehyde, sectioned at 5 μm, and subsequently stained with H&E (for RV hypertrophy) or Masson’s trichrome (for fibrosis). Cardiomyocyte cross-sectional area was obtained by tracing the outlines of cardiomyocytes with a clear nucleus image in H&E-stained images. The quantification of fibrosis and cross-sectional area of cardiomyocytes were determined in at least ten randomly chosen areas with ImageJ software^54^.

### Right ventricle tissue isolation, library preparation and RNA-seq

Human RV tissues were manually dissected in cardiac biopsy or autopsy, according to the protocol previously described^20^. RV tissues from MCT-treated, and PAB-induced rats were harvested after hemodynamic measurements and snap frozen for further assessment. Total RNA from each batch of RV samples was isolated separately using the RNeasy Mini Kit (miRNeasy Micro Kit, when less sample provided; Qiagen) and snap frozen. Purification of total RNA for both human and animal tissues was performed, as described in the RNeasy handbook, using DNase I digestion (RNase-free DNase Set, Qiagen). RNA integrity was verified using a LabChip GX Touch 24 (PerkinElmer). Approximately 10 ng and 2 µg of total RNA, respectively, were used as starting material for library preparation using the SMARTer Stranded Total RNA-seq Kit - Pico Input Mammalian (Takara Bio), and the VAHTS Stranded mRNA-seq Library Prep Kit (Vazyme), following the manufacturers’ protocols. Sequencing was performed using the NextSeq 500 platform (Illumina) with paired-end setup (first batch of human RV tissues) and 75-bp single-end setup (the rest of datasets).

### Bulk RNA-seq data analysis

Raw reads were assessed for quality, adaptor content and duplication rates using FastQC^55^. Trimmomatic version 0.39 was used to trim reads following a quality drop (below a mean of Q15) in a window of five nucleotides^56^. Trimmed and filtered reads between 15 and 150 nucleotides were cleared for further analysis and aligned against the Ensembl Human Genome (version hg38; GRCh38.27) and Rat Genome (version Rn06) using STAR software version 2.6.1 to include multi-mapping reads, with the parameters: outFilterMismatchNoverLmax, 0.1; outFilterScoreMinOverLread, 0.9; outFilterMatchNminOverLread, 0.9; alignIntronMax, 200,000; and outFilterMultimapNmax, 999 (ref. ^57^). The number of reads that aligned to genes was counted using ‘featureCounts’ version 1.6.5 from the ‘Subread’ package^58^. Only reads mapping at least partially inside exons were aggregated and counted per gene. Reads aligning to multiple regions or genes were excluded.

Raw count values for each organism were normalized separately using DESeq2 (version 1.30.0)^59^, then transformed into regularized logarithm values for further analysis. Because we had two different batches of human samples, we performed a batch effect correction on this data using the sva package^60^. The normalized and batch-corrected data were the basis of downstream analysis.

DEGs were identified using DESeq2 (ref. ^59^). Those with a Benjamini–Hochberg-corrected *P* value ≤ 0.05 were considered to be upregulated. Those with log_2_FC ≥ 0.58 and ≤ −0.58 were considered to be downregulated. The annotations were enriched with UniProt data (release 25.06.2019), based on Ensembl gene identifiers (activities at the Universal Protein Resource).

Dimension reduction analyses (PC analyses) were performed on normalized and regularized, log-transformed DESeq2 counts using the PC analysis and FactoMineR packages for R software (Foundation for Statistical Computing). Heatmaps were generated, using the ‘complexheatmap’ R package, as visualizations of the most differentially regulated genes. For assembly of the heatmap, normalized count values of all DEGs were transformed by a *z*-score transformation per row. Volcano plots were produced to show DEG regulation, per contrast, based on DESeq2 normalized counts.

Unsupervised clustering for 40 RV samples from human and for 26 MCT-induced rat samples was performed using *k*-means clustering. *k*-means clustering is a well-known, and widely used unsupervised machine learning algorithm used to partition a dataset into distinct groups or clusters based on their similarities. It starts by randomly initializing cluster centroids and assigns data points to the nearest centroid. The centroids are then updated iteratively by recalculating their means based on the assigned data points, and the process continues until convergence. The algorithm aims to minimize the within-cluster sum of squared distances, producing a final clustering solution. *k*-means clustering is an efficient method; however, since its results can be sensitive to initialization parameters, it requires multiple runs to ensure robustness. Therefore, we tested different values of *k* on normalized counts to optimize the clustering for each dataset. After comparing the possibilities, with the optimized *k* = 4 for rat samples and *k* = 5 for human samples were selected for further analysis. Second-step DEG analysis was performed using DESeq2 with the same criteria as in the first step, in which we initially performed DEGs between all identified clusters (clusters B–E for human, and B–D for MCT rat RVs) versus control samples from cluster A. Furthermore, we performed a comparison between subgroups based on their RV state (compensated versus decompensated), considering their different clusters. Finally, DEG analysis was performed for some extra pairs of comparisons between identified clusters.

All groups of DEG lists were submitted to gene-set enrichment analyses using KOBAS 3 (ref. ^61^). The resulting bar plots show Gene Ontology terms and KEGG pathways, with corrected FDR < 0.05 considered to indicate significantly deregulated pathways, for each pair of comparisons in both directions. The numbers of under-represented and over-represented genes are shown along with each pathway.

Combinatorial heatmaps visualize all the specific deregulated genes, in some of the common, significantly altered pathways, in the human and rat datasets. All samples were distributed unsupervised through the *x* axis of the heatmap, which has been highlighted by the identified subgroups. Violin plots were generated using r-log-normalized count values for all samples, divided into subgroups along the *x* axis. When the differentially regulated pathways were identified and summarized in both datasets, we used a generalized additive model to represent the relative expression of corresponding genes in each selected pathway, through RV states that ranged from normal to late decompensated.

### Metadata analysis/methods

Using participant background metadata and the whole gene expression profile, we used a simple generalized linear regression (using ‘glmnet’ R package^62,63^) to first find binominal genes (184), and then determine whether the assigned expressions could be predicted by (or correlated with) any of the given metadata (batch, sex, age and sample type). In addition, leave-one-out cross-validation was used to evaluate the performance of the model. Of the 184 bimodal genes, only 49 genes demonstrated AUC greater than 0.6, indicating batch as the most influential predictor among the selected variables, while the participant background differences such as age/sex did not have a significant effect. We also used standard error estimation (‘glmnet’ R package) to calculate the accuracy of prediction for the selected genes, which resulted in error rate ≥ 0.5 for most of them. This implies a very high misclassification error, which is mainly due to the small sample size per group/type, as it becomes unavoidable in our study with 40 participant samples.

### Gene expression quantification by real-time quantitative PCR

Real-time quantitative PCR analyses were performed as previously described^64^. Briefly, total RNA from cells/tissues and plasma were extracted using TRIzol (Invitrogen), according to the manufacturer’s instructions. Total RNA was reverse-transcribed into complementary DNA and qPCR was performed, using appropriate primers (all the oligonucleotide sequences are provided in (Supplementary Tables 1 and 2). Glyceraldehyde 3-phosphate dehydrogenase or 18 S ribosomal RNA were used as housekeeping genes for analysis. The relative expression levels of each candidate gene were determined by the ΔC_t_ method. Values are expressed as means ± s.e.m. The Shapiro–Wilk normality test was used to determine whether the collected data was normally distributed. Comparisons of means between two groups used the unpaired *t*-test, for normally distributed samples, or the Mann–Whitney *U* test, for non-normally distributed samples. Comparisons between means among three or more groups were performed by one-way ANOVA for normally distributed samples, followed by Tukey’s multiple-comparisons test.

### Western blotting and quantification

RV tissues were homogenized in RIPA lysis buffer (Thermo Scientific), quantified and lysates were separated on 7% and 10% polyacrylamide gels and transferred to PVDF membranes. After blocking, the membranes were probed with corresponding primary antibody overnight at 4 °C. This was followed by 2 h of incubation with secondary antibodies conjugated with horseradish peroxidase and protein levels were detected by chemiluminescence with the SuperSignal West Femto substrate solution (Thermo Scientific). The image was developed using iBright Image reader (Thermo Scientific), and densitometric analysis of the blots was obtained using Fiji ImageJ. Expression was quantified using band intensity values (in arbitrary units), which were normalized to vinculin. A list of the antibodies used in this study is provided in (Supplementary Table 3).

### Plasma sample collection

For the German cohort (discovery), blood samples from 35 IPAH participants (male and female) participating in the ‘Right Heart 1 trial’ (NCT03403868) were collected at University Hospital Giessen and Marburg, Department of Pneumology and Critical Care Medicine (Germany) from 2016 to 2018. Fasting blood samples were obtained during RHC and immediately frozen at −80° for the following proteomic assay. All data collection and experimental procedures were performed with the approval of the local ethics committee (AZ 58/15).

For the UK cohort (served as validation), blood samples from 61 PAH participants (male and female) undergoing RHC were obtained at Sheffield Pulmonary Vascular Unit (United Kingdom) from March 2013 to February 2018. All experimental procedures were performed with the approval of Laval University and the IUCPQ Biosafety and Ethics Committees (CER 20735), and the Sheffield Teaching Hospitals Observational Study of Pulmonary Hypertension, Cardiovascular and other Respiratory Diseases Scientific Advisory Board (UK REC ref. 18/YH/0441), with support from the NIHR Sheffield Clinical Research Facility. Fasting blood samples were collected following RHC measurements in both cohorts of study, and immediately frozen at −80° until proteomics analysis.

### Characterization of pulmonary arterial hypertension participants in two proteome cohorts

Similar to the transcriptome cohort, participants who were not diagnosed with any cardiac or respiratory diseases were selected as the control group in the validation proteome cohort. The discovery cohort does not contain any control group. PAH participants from the discovery cohort were characterized using the coupling ratio of Ees/Ea as previously described^19^. In brief, participants with Ees/Ea ≥ 0.8 were considered as those with a compensated RV state, while Ees/Ea < 0.8 defined the worsening RV condition and considered as participants with a decompensated RV state. In the validation cohort, primary characterization was performed based on CI value; participants with compensated RV identified with CI > 2.2 l min^−1^ per m^2^ and decompensated RV with CI < 2.2 l min^−1^ per m^2^. Normal distribution of age and sex per group of participants (compensated to decompensated) was tested in both cohorts using Welch two-sample *t*-test for age, and Fisher’s exact test for sex, to confirm that there were no significant effects of these two factors in any of the comparisons.

### Plasma proteome assay and analysis

Proteome assays were performed using a high-throughput multiplex proximity extension assay technology (Olink Bioscience) as previously described^65^. The exploration cohort was generated by cardiometabolic protein panel, version 3601 (including 92 proteins), and for the second cohort, a 384-plex panel (including 372 proteins) focusing on cardiometabolic proteins was used. The assay utilizes epitope-specific binding and hybridization of a set of paired oligonucleotide antibody probes, which is subsequently amplified using a qPCR, resulting in log_2_-normalized protein expression (NPX) values where a high value corresponds to a higher protein expression^65^. Statistical analysis to identify differentially regulated proteins was performed using the unpaired *t*-test between two groups (German cohort), for normally distributed samples, and among three groups (validation cohort) by one-way ANOVA, followed by Tukey’s post hoc test for multiple-comparisons correction. For the exploration cohort, no correction was used for *P* values to extend the protein selection for further confirmation using a validation cohort.

Correlations of different functional RV parameters with selected protein expression as well as proBNP levels were assessed by a simple linear regression model (for a single variable), and multiple regression model (when correction for age/sex was considered). Regression analyis *P* values were reported for each single comparison.

### Random forest model and importance score measurement

We used a random forest regression-based model to test subgroup classification performance by the panel of five protein expression levels. We first ran the model on each PAH cohort independently for all the five proteins together, and then for different combinations. Random forest model testing, along with the tenfold cross-validation method to avoid overfitting, was implemented using the R package ‘ranger’^66^. Then, ROC AUC was used to assess the random forest model performance respective to each classification/combination. Corresponding ROC *P* values were calculated using a Mann–Whitney (Wilcoxon) test for the H0 = ‘the AUC is equal to 0.5’, which is implemented in R package ‘verification’ (https://cran.r-project.org/web/packages/verification/index.html)^67^.

Furthermore, we used the permutation approach to measure variable importance (in this case, features are proteins) using the ‘ranger’ package. This accuracy-based approach enabled us to calculate the importance of a specific variable using an out-of-bag estimation method. In brief, the importance score is defined as the difference between the calculated prediction accuracy and the prediction accuracy while the measured variable is randomly shuffled and all other variables remain the same.

### Survival curve analysis

Transplant-free survival was analyzed from sampling to death or lung transplantation. The cutoff date was 30 September 2021. ROC curves were constructed for protein alone or several proteins to assess the optimal cutoff value and their performance in predicting transplant-free survival. Logistic regression models were used to calculate the combined models using several proteins for ROC analyses. The Kaplan–Meier analysis was used to estimate transplant-free survival rate, and a log-rank test was used for comparisons between groups using the optimal cutoff point with the ROC curves. For survival curves using several proteins, in each participant, each protein counted as one if the protein was greater than or equal to the level of the cutoff. For each participant, the numbers of selected proteins that were equal to the cutoff level or more than the cutoff level was summed, and participants were divided into the groups of the summed number. A significance level inferior to 5% (*P* < 0.05) was considered statistically significant.

All statistical analyses were made with R version 4.0.3 (R Foundation for Statistical Computing), or SPSS version 27 (IBM), or Prism version 9.4.0 (GraphPad).

### Reporting summary

Further information on research design is available in the Nature Portfolio Reporting Summary linked to this article.

## Supplementary information

Supplementary Information Supplementary Figs. 1–11 and Tables 1–3.

Reporting Summary

Supplementary Data 1 List of DEGs and enriched pathways in rat RV from MCT-induced PH.

Supplementary Data 2 List of DEGs and enriched pathways in rat RV from PAB model.

Supplementary Data 3 Binominal genes predicted by metadata factor analysis (linear regression model).

## Source data

Source Data Fig. 3 Clinical and echocardiographic characteristics of human RV transcriptome cohort. List of DEGs and enriched pathways in human RV transcriptomic. Comparison of decompensated RV DEGs in MCT, and PAB rats and human PAH datasets.

Source Data Fig. 4 Female and male inclusion in human RV transcriptome cohort. List of DEGs and enriched pathways in male versus female human RV conditions. Hemodynamics characterization of male and female MCT-induced rats (second batch). List of DEGs and enriched pathways in male versus female MCT-induced rat RVs.

Source Data Fig. 5 List of DEGs and enriched pathways in MCT-induced rat RV subgroups.

Source Data Fig. 6 List of DEGs and enriched pathways in human RV clusters. Collective Gene Ontology terms and their associated DEGs in human RV. List of DEGs and enriched pathways in human RV subgroups.

Source Data Extended Data Fig. 1 Hemodynamic characterization in male MCT-induced rats (first batch).

Source Data Extended Data Fig. 2 Hemodynamics characterization in male PAB rat.

Source Data Extended Data Fig. 3 List of final target genes associated with regulation of hypoxia, cell cycle and ECM remodeling.

Source Data Extended Data Fig. 4 Uncropped scan of western blot gels 1 and 2.

Source Data Extended Data Fig. 4 Public proteomic evidence used for validation and correlation of target genes.

Source Data Extended Data Fig. 5 Patients characterization of German PAH plasma proteome cohort (discovery cohort).

Source Data Extended Data Fig. 6 Patients characterization of UK PAH plasma proteome cohort (validation cohort).

Source Data Extended Data Fig. 7 Uncropped scan of western blot gels 1 and 3.

## Data Availability

All the expression data (human and MCT and PAB rat RV) generated in this study are deposited in the Gene Expression Omnibus under accession number GSE240941. Source data are provided with this paper including the clinical data of all the participants in transcriptome and plasma proteome cohorts. All other data supporting the findings in this study are available within the article or Supplementary Information.
