# Supplementary material for: Transcriptional profiling unveils molecular subgroups of adaptive and maladaptive right ventricular remodeling in pulmonary hypertension
Source: Nat Cardiovasc Res. 2023 Sep 28;2(10):917–36. doi: 10.1038/s44161-023-00338-3 (PMC11358157; doi:10.1038/s44161-023-00338-3)
Supplement: Supplementary file 1 — Supplementary Figs. 1–11 and Tables 1–3. [file 44161_2023_338_MOESM1_ESM.pdf]

nature cardiovascular research

Article

https://doi.org/10.1038/s44161-023-00338-3

Transcriptional profiling unveils molecular subgroups of adaptive and maladaptive right ventricular remodeling in pulmonary hypertension

In the format provided by the authors and unedited

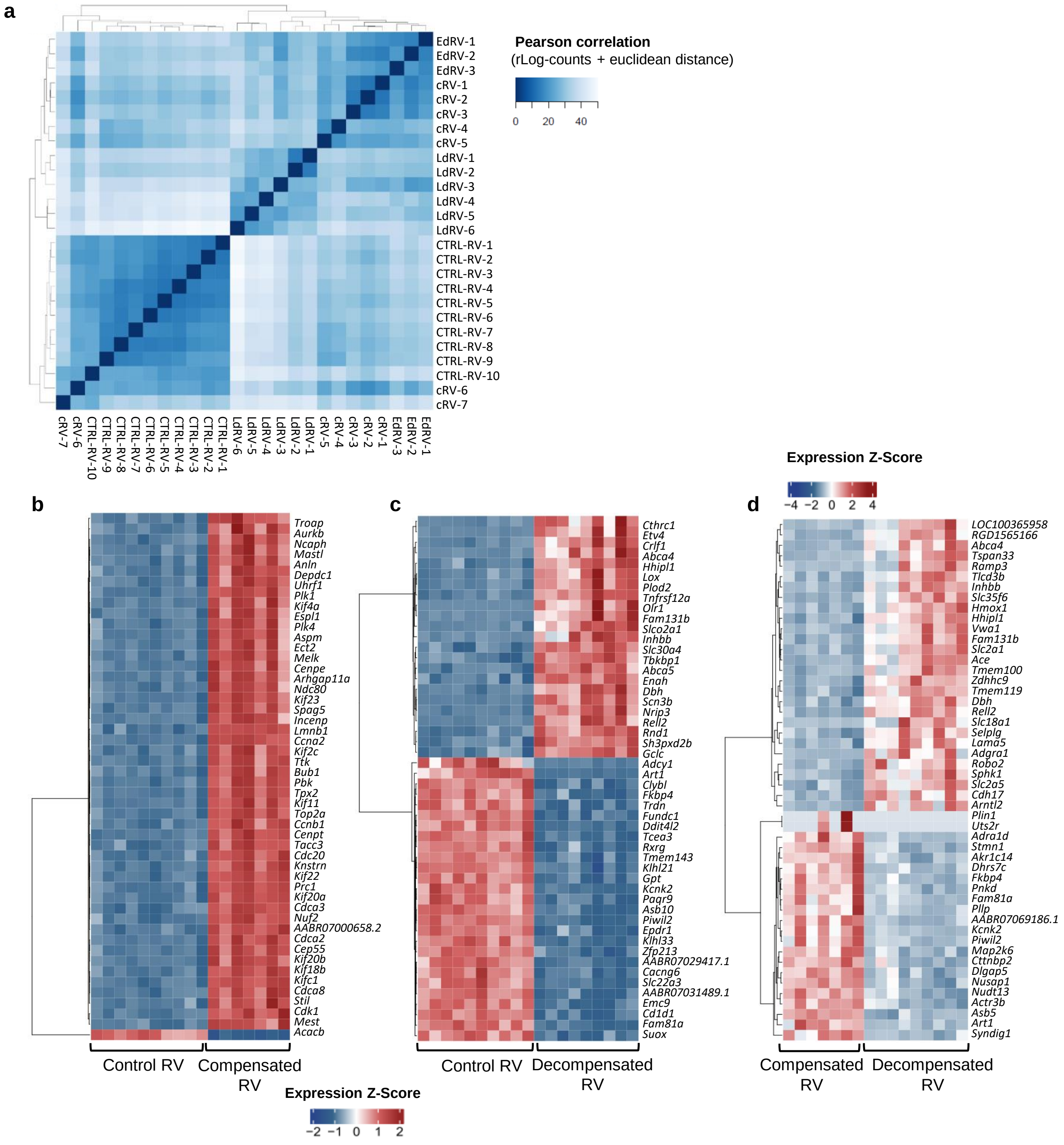

Supplementary Figure 1: Gene expression correlation between all MCT-rat RV samples and top-50 DEG in each pair. (**a**) Pearson correlation heatmap for normalized couts of all the RV samples from MCT-rat shows high in-group similarity, also confirms the similarity of three early decompensated samples within the compensated group (control (n)=10, compensated (n)=7, decompensated (n)=9). (**b-d**) Heatmap representation of top 50 significant DEGs (basemean expression ≥5; −0.585 ≤log2FC≥ 0.585; FDR ≤0.05) for three pairs of comparisons. Scaled z-scores of normalized counts are shown.

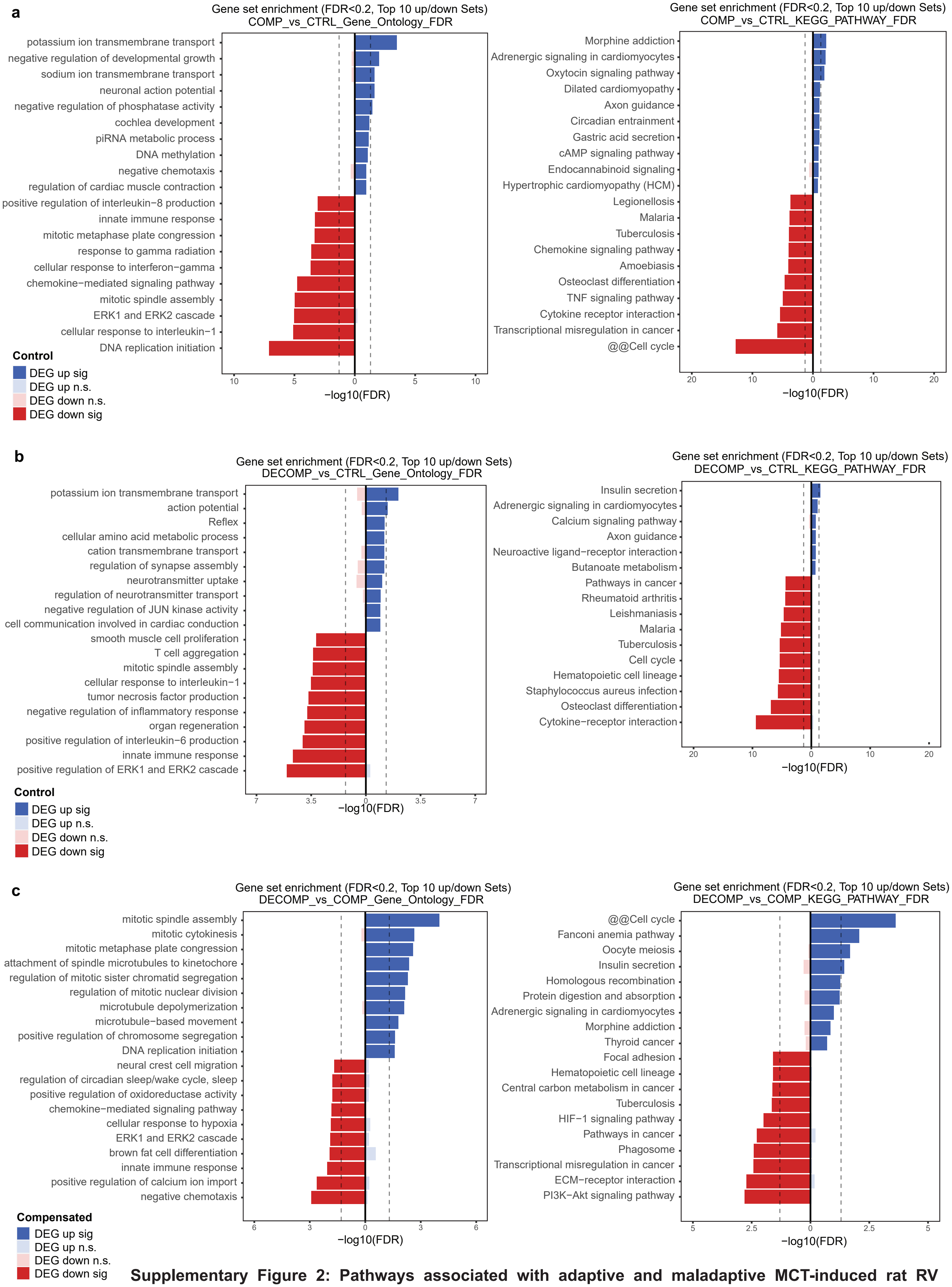

Supplementary Figure 2: Pathways associated with adaptive and maladaptive MCT-induced rat RV remodeling. Pathway enrichment analysis of significant DEGs from MCT-induced PH. The Gene Ontology and KEGG pathways that are differentially enriched in (**a**) compensated vs control RV, (**b**) decompensated versus control RV, and (**c**) compensated versus decompensated RVs. The dashed line indicates FDR=0.05.

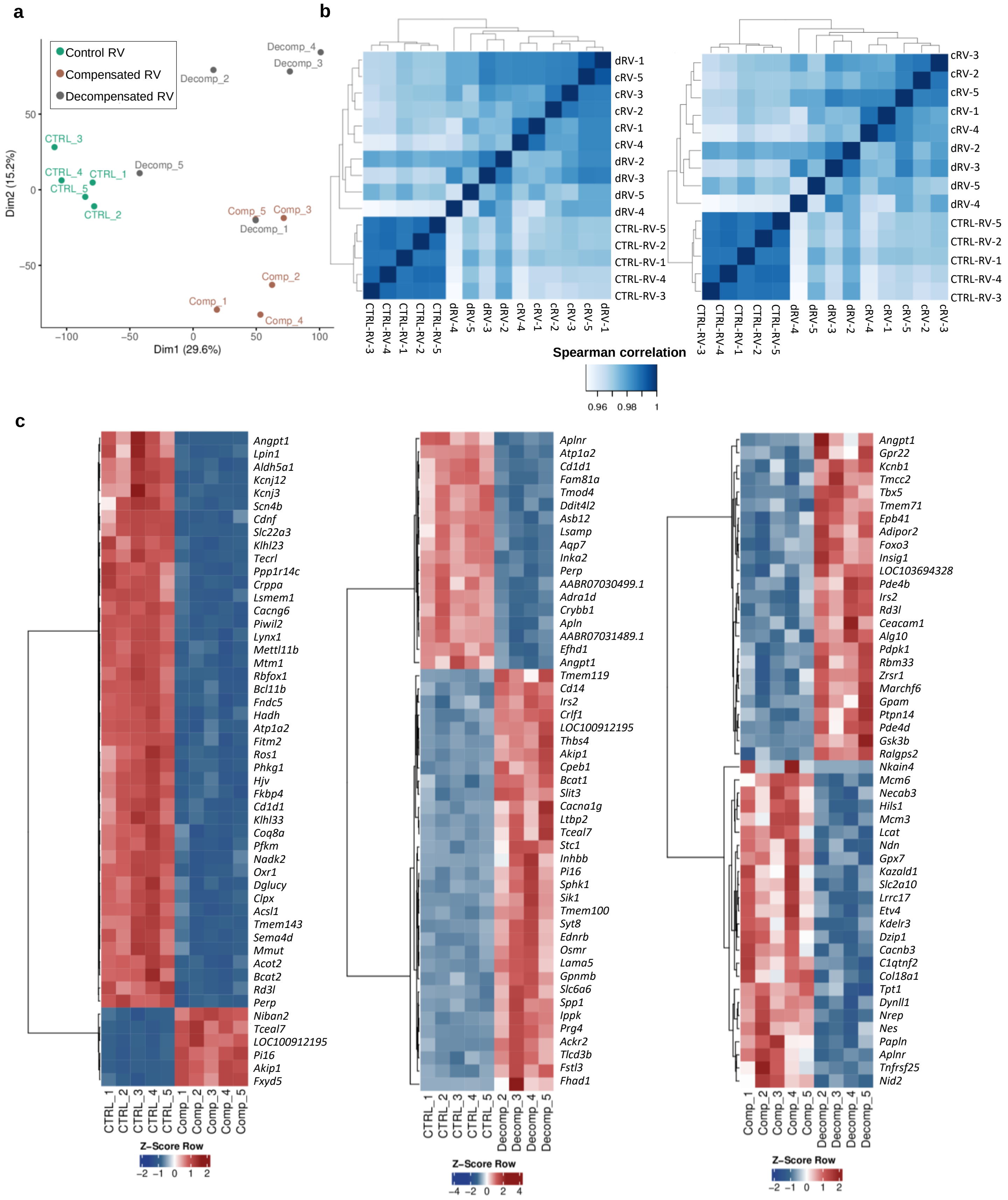

Supplementary Figure 3: Gene expression correlation between all PAB rat RV samples and top-50 DEG in each pair. (**a**) PCA plot representing all 15 PAB-rats RV samples. One decompensated sample (Decomp_1) has been removed from downstream analysis due to lower similarity with the rest of decompensated samples. (**b**) Spearman correlation heatmap for all the RV samples, which shows decompensated-1 as outlier (right) and for 14 RV samples after removing one decompensated shows high in-group similarity (control(n)=5, compensated(n)=5, decompensated(n)=5). (**c**) Heatmap representation of the 50 most significant DEGs (basemean expression ≥5; −0.585 ≤log2FC≥ 0.585; FDR ≤0.05) for three pairs of comparisons. Scaled z-scores of normalized counts are shown.

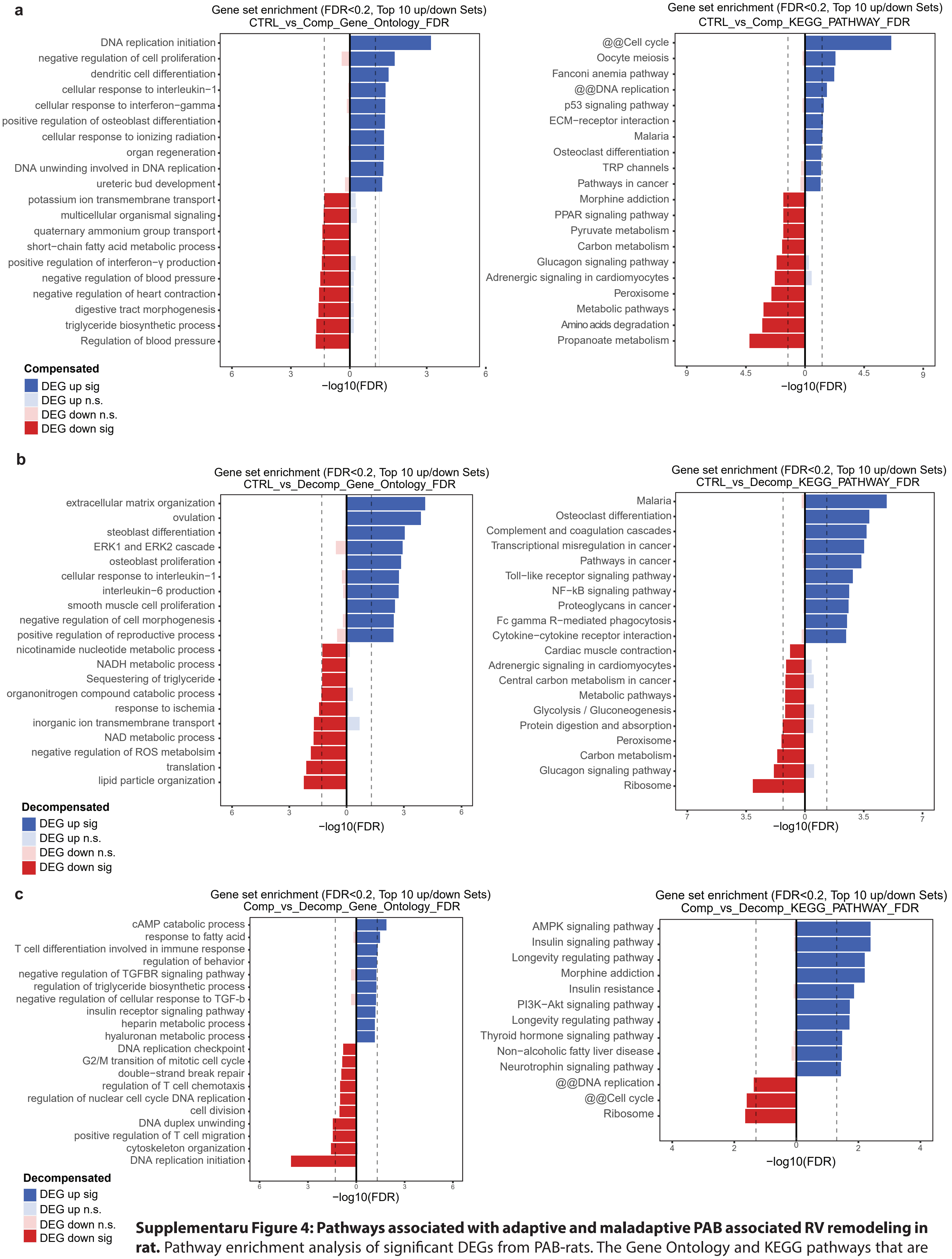

Supplementaru Figure 4: Pathways associated with adaptive and maladaptive PAB associated RV remodeling in rat. Pathway enrichment analysis of significant DEGs from PAB-rats. The Gene Ontology and KEGG pathways that are differentially enriched in (**a**) compensated vs control RV, (**b**) decompensated versus control RV, and (**c**) compensated versus decompensated RVs. The dashed line indicates FDR=0.05.

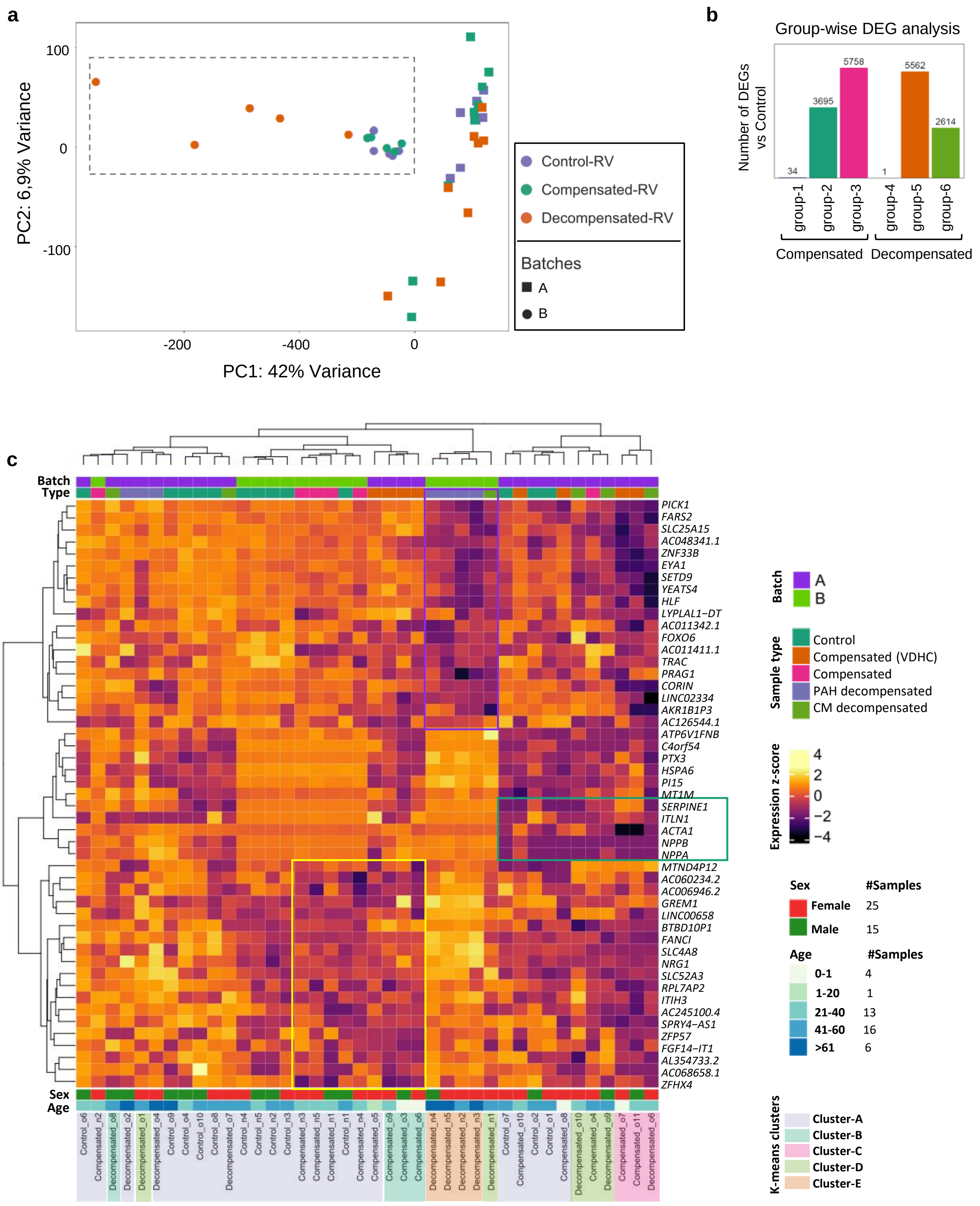

Supplementary Figure 5: Meta-analysis of human samples based on patients' clinical information. (**a**) PC analysis show the human dataset before batch effect normalization. (**b**) Group-wise comparisons within each conditions versus control. Each subgroup (represented by different colors) includes samples from the same cluster and only from the same condition. The numbers of genes with basemean ≥5; −0.585≤log2FC≥0.585; FDR≤0.05 are shown. (**c**) The heatmap shows 49 genes with AUC > 0.6 from the linear regression model analysis, which show the highest correlation with different batches of samples. K-means colors has been added to this figure after applying the clustering method on the dataset, which shows three smaller subset of genes with high correlation with compensated and decompensated subgroups.

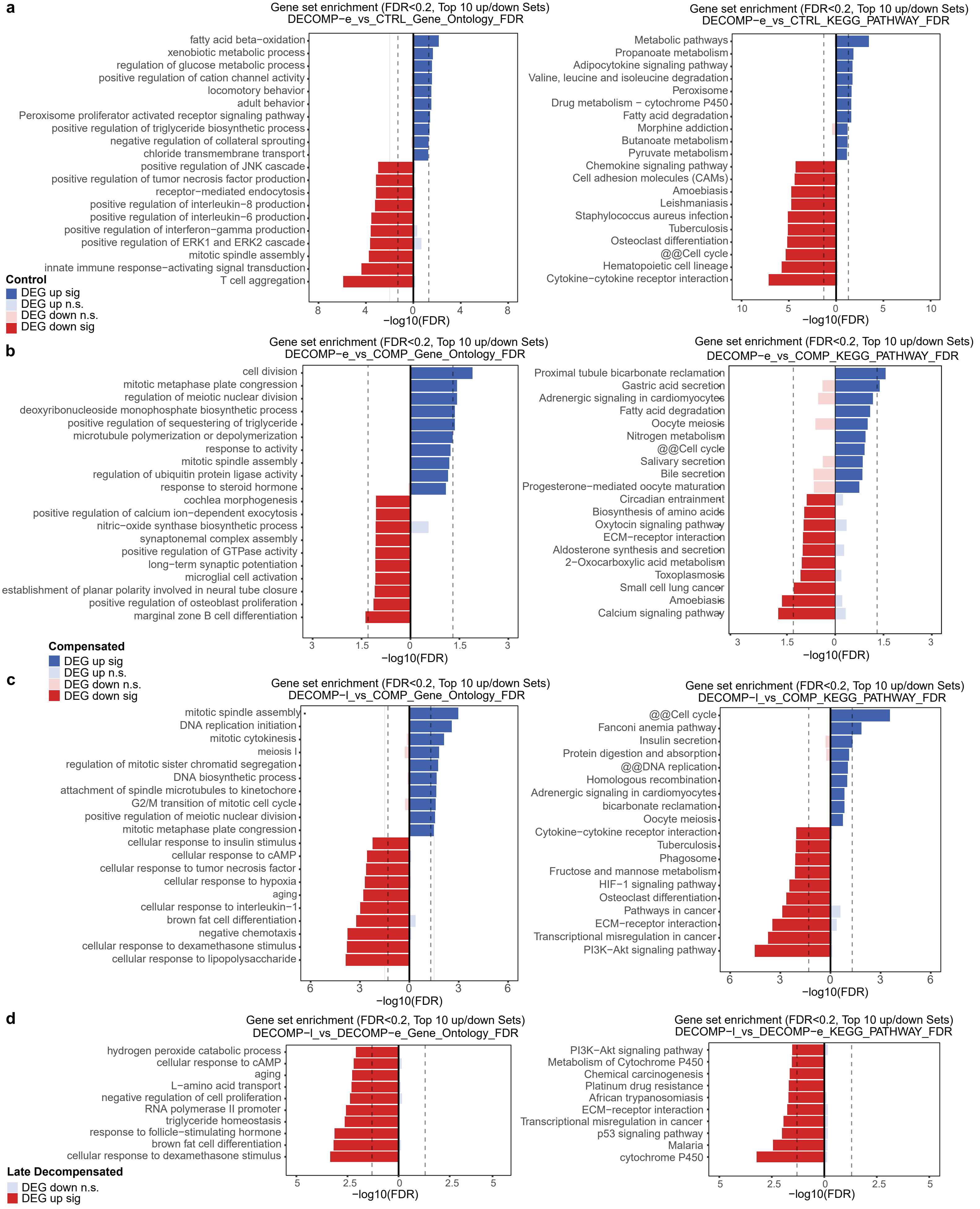

Supplementary Figure 6: Altered pathways in early and late decompensated MCT-induced PH. Top gene ontologies (GO) and KEGG pathways differentially regulated in (**a**) early decompensated versus control RV, (**b**) early decompensated versus compensated RV, (**c**) late decompensated vs compensated RV , and (**d**) late vs. early decompensated RV in MCT-induced rats. The dashed line indicates FDR=0.05.

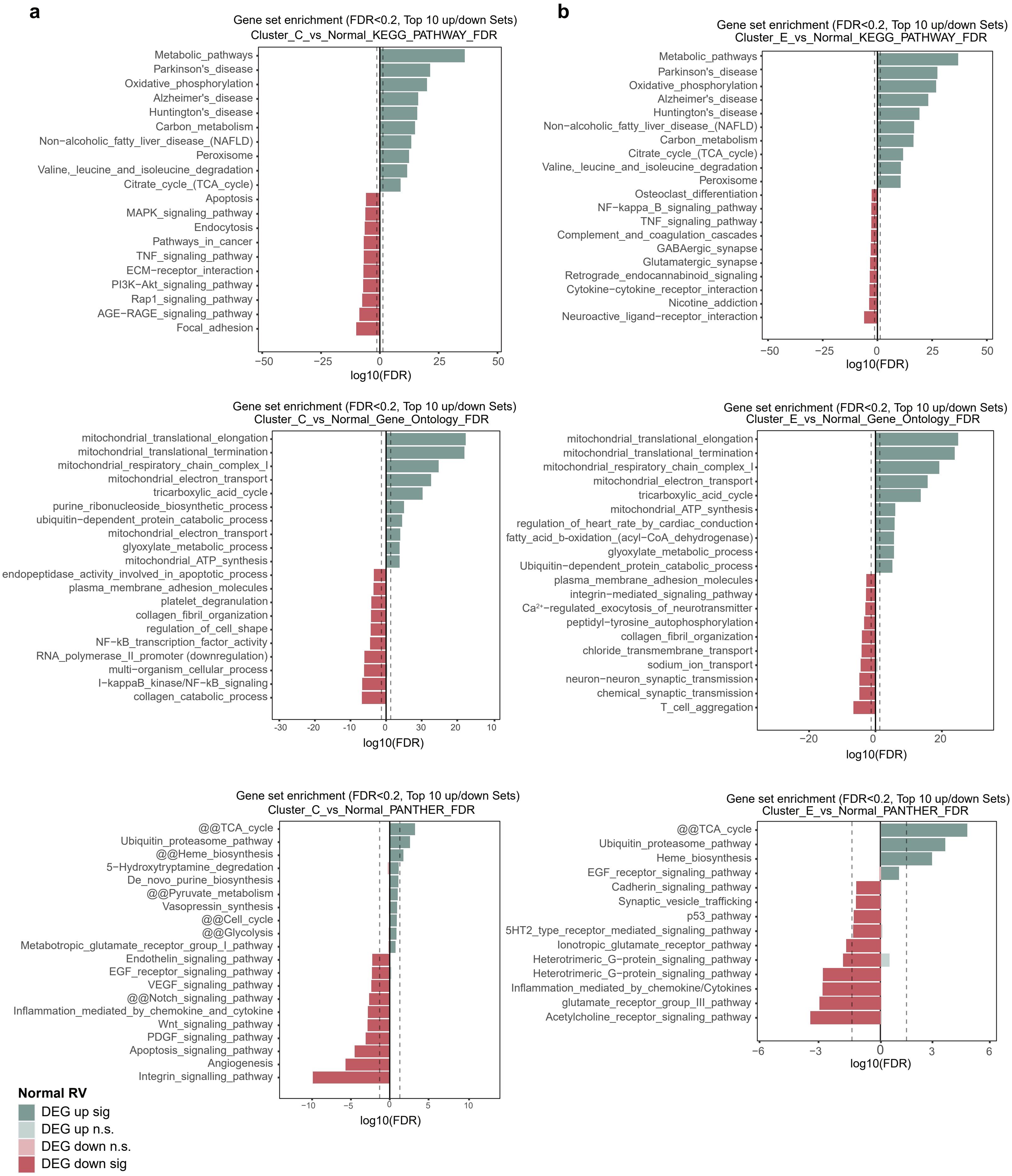

Supplementary Figure 7: Pathways associated with subgroups from human compensated and decompensated RV. Top KEGG pathways, gene ontologies (GO), and PANTHER pathways differentially enriched in (**a**) cluster C versus normal RV, and (**b**) cluster E versus normal RV. The dashed line indicates FDR=0.05.

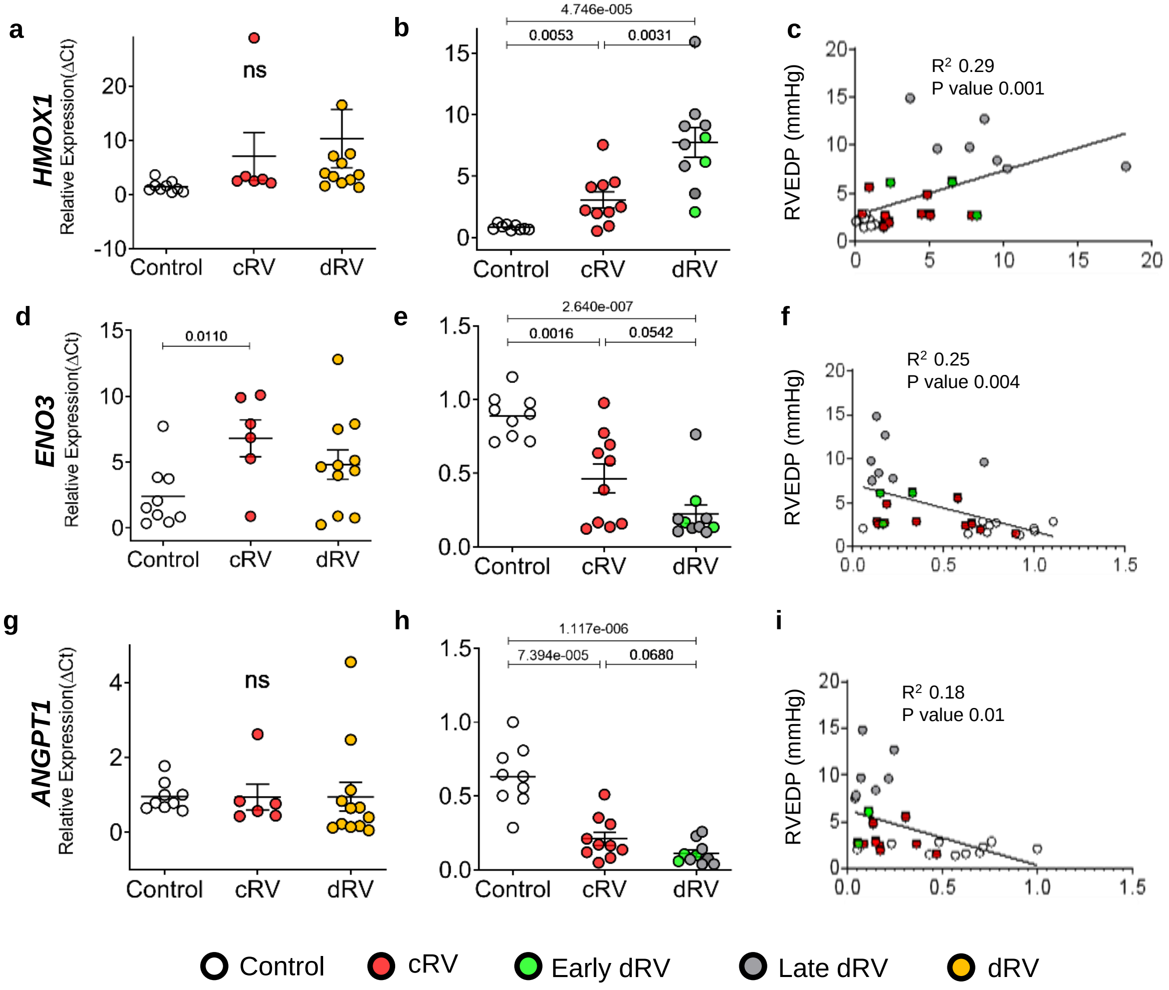

Supplementary Figure 8: Validation of common differentially expressed genes in right ventricle tissues from human and rodent PH. The relative expression levels of the candidate genes *HMOX1, ENO3*, and *ANGP1* were analyzed by qPCR in RV samples from human and rat MCT-induced PH. **(a, d, g)** qPCR analysis of candidate genes from human control, compensated, and decompensated RVs, and **(b, e, h)** from compensated, and decompensated RVs isolated from rat MCT-induced PH and respective controls. **(c, f, i)** Correlation between expression levels of target genes and right ventricular end-diastolic pressure. (RVEDP= right ventricle end diastolic pressure). Data are presented as mean ± SEM. (Expression levels has been tested by unpaired t-test, and *p-value* has not been corrected for multiple comparison as it does not apply). (a,d,g): n(normal)=9, n(compensated)=6, n(decompensated)=12. (b,e,h): n(normal)=10, n(compensated)=10, n(decompensated)=10

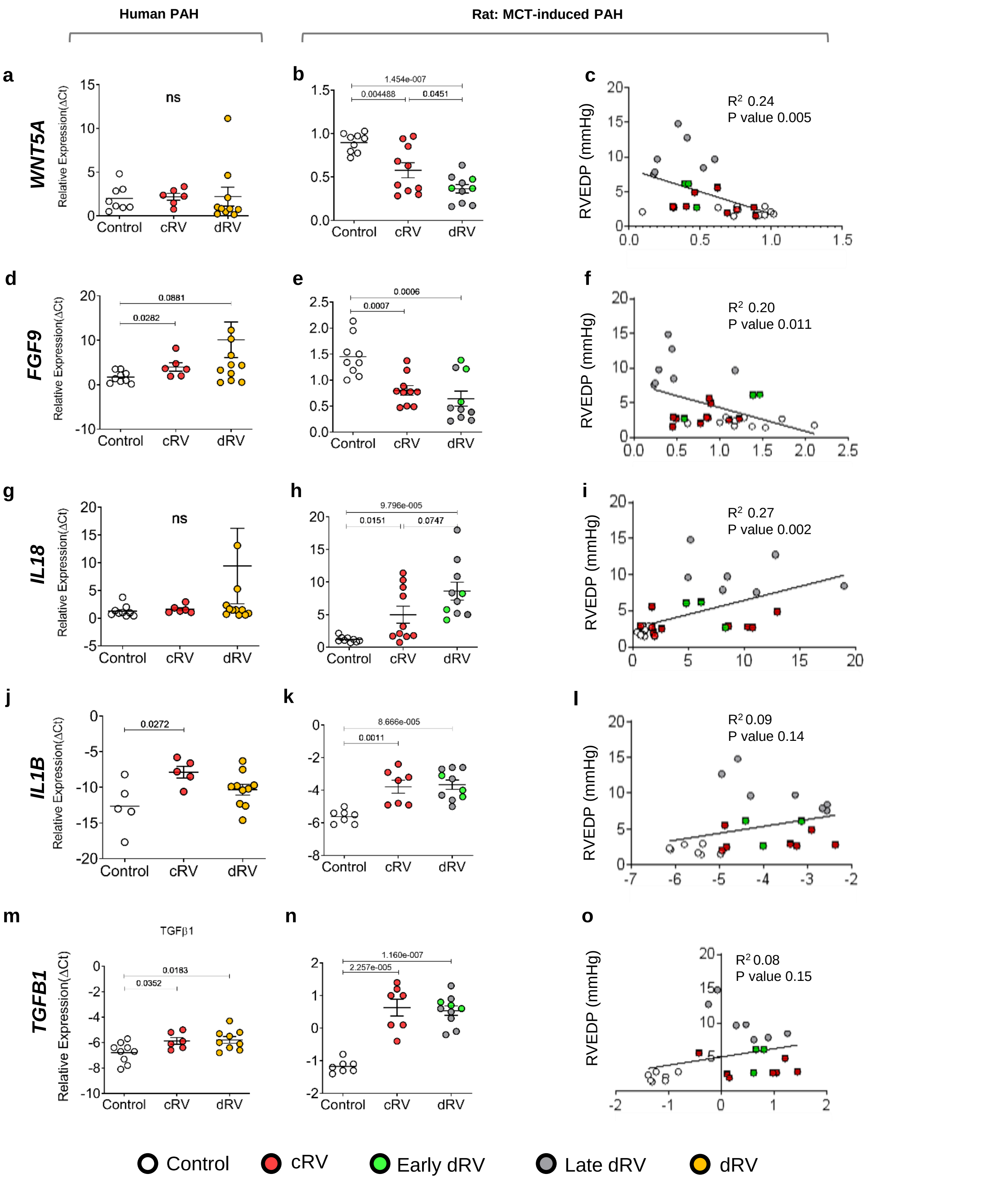

Supplementary Figure 9: Validation of common differentially expressed genes in right ventricle tissues from human and rodent PH. The relative expression levels of the candidate genes: *WNT5A, FGF9, IL18, IL1B*, and *TGFB1* were analyzed by qPCR in RV samples from human and rat MCT-induced PH. (**a, d, g, j, m**) qPCR analysis of candidate genes from human control, compensated, and decompensated RVs, and (**b, e, h, k, n**) from compensated, and decompensated RVs isolated from rat MCT-induced PH and respective controls. (**c, f, i, l, o**) Correlation between expression levels of target genes and right ventricular end-diastolic pressure. (RVEDP= right ventricle end diastolic pressure). Data are presented as mean ± SEM. (Expression levels has been tested by unpaired t-test, and *p-value* has not been corrected for multiple comparison as it does not apply). n(normal-d,g,m)=9, n(normal-a)=8, n(normal-j)=5, n(compensated-a,d,g,m)=6, n(compensated-j)=5, n(decompensated-a,j)=10, n(decompensated-d,g)=12, n(decompensated-m)=9. (b,e,h,k,n): n(normal)=10 (k,n:7), n(compensated)=10 (k,n:7), n(decompensated)=10

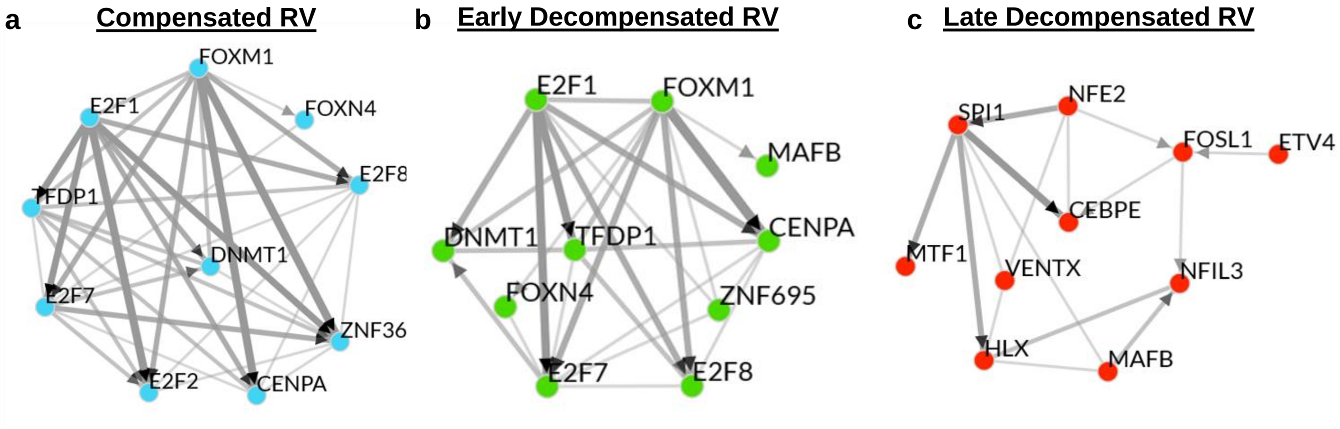

Supplementary Figure 10: Transcription factors enrichment analysis for RV subgroups in MCT-rats. Top 10 enriched transcription factors in each pair of comparisons demonstared in their respective protein-protein interaction network; compensated versus control (**a**), early decompensated versus control (**b**), and late decompensated versus control (**c**) in MCT-induced PH.

ECM Regulation

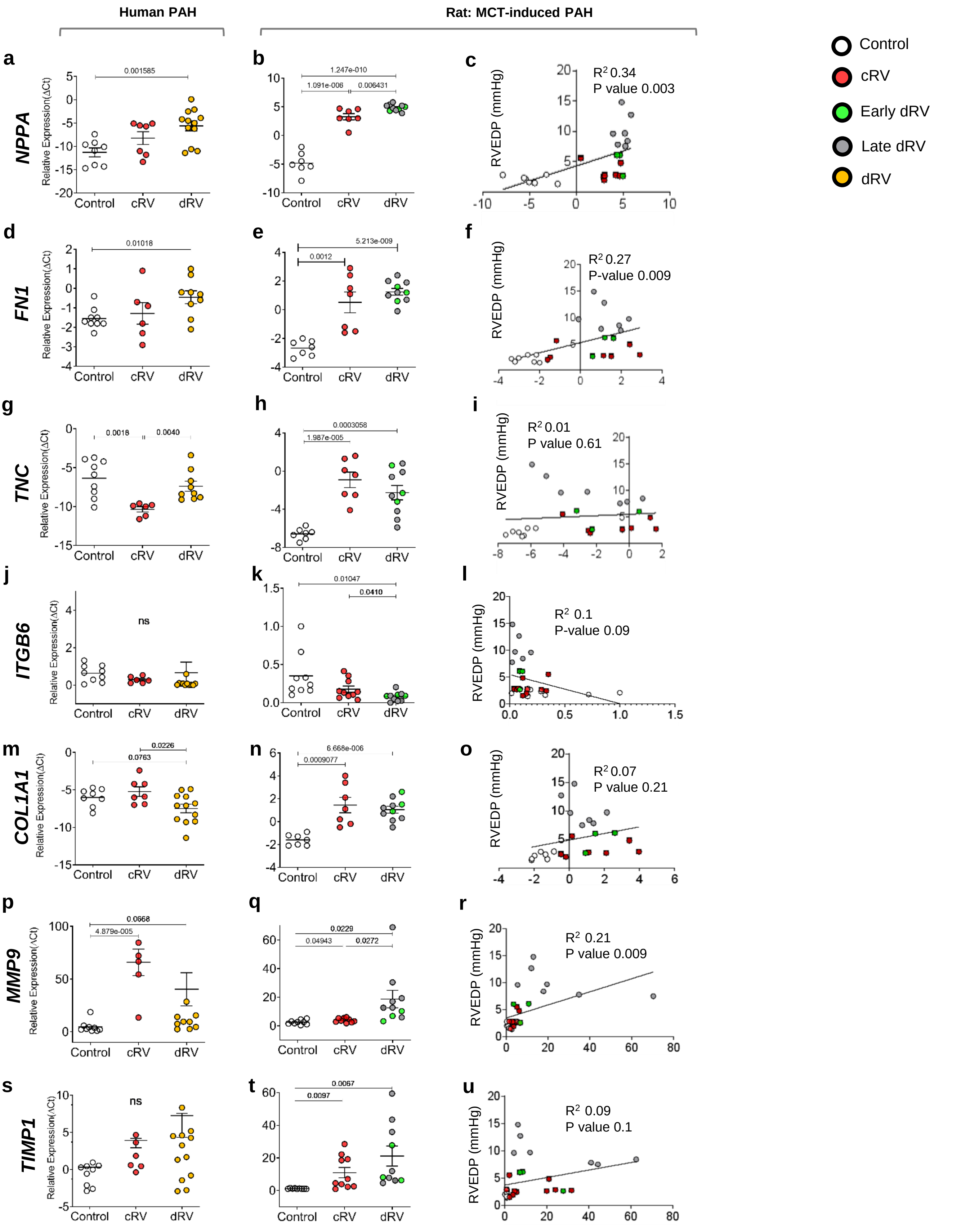

Supplementary Figure 11: Validation of common differentially expressed genes in right ventricle tissues from human and rodent PH. The relative expression levels of the candidate genes *NPPA, FN1, TNC, ITGB6, COL1A1, MMP9* and *TIMP1* were analyzed by qPCR in RV samples from human and rat MCT-induced PH. (**a, d, g, j, m, p, s**) qPCR analysis of candidate genes in cDNA samples reverse-transcribed from RNA isolated from human control, compensated, and decompensated RV tissues, as well as control and MCT-induced RVs from rat (**b, e, h, k, n, q, t**). (**c, f, i, l, o, r, u**) Correlation between expression levels of target genes and right ventricular end-diastolic pressure. (RVEDP= right ventricle end diastolic pressure). Data are presented as mean ± SEM. (Expression levels has been tested by unpaired t-test, and *p-value* has not been corrected for multiple comparison as it does not apply). n(normal-d,g,j,p,s)=9, n(normal-a)=7, n(normal-m)=8, n(compensated-a,m)=7, n(compensated-d,g,j,p,s)=6, n(decompensated-d,g,j)=9, n(decompensated-a,m,p,s)=12. n(normal-b,e,h,n)=7, n(normal-k,q,t:10), n(compensated-b,e,h,n)=7, n(compensated-k,q,t)=10, n(decompensated=10 (k:9))

Supplementary Table 1- Human Primers

Gene name	Primer Sequence
CCL2_FW	CAGCCAGATGCAATCAATGCC
CCL2_RV	TGGAATCCTGAACCCACTTCT
IL1B_FW	AGCTACGAATCTCCGACCAC
IL1B_RV	CGTTATCCCATGTGTCGAAGAA
TGFB1_FW	CGACTCGCCAGAGTGGTTAT
TGFB1_RV	CGGTAGTGAACCCGTTGATGT
FOXM1_FW	ATTGCCCGAGCACTTGGAATCA
FOXM1_RV	CTCAGCTAGCAGCACCTTG
FN1_FW	AGCAGACCCAGCTTAGAGTT
FN1_RV	GCAGAAGTGTTTGGGTGACT
NPPA_FW	TGTCCAACGCAGACCTGATG
NPPA_RV	AGGGCAGATCGATCAGAGGA
TNC_FW	CCAATCATTTGAACAAAGCGGG
TNC_RV	TGTAAGCTTTTCCCAAGTGTGT
COL1A1_FW	ACATGTCTAGGGTCTAGACATGTTC
COL1A1_RV	ACCTTGCCGTTGTCGCAGAC
ENO3_FW	CCCTGACCTCATACTCCCA
ENO3_RV	AGGCAGAATCATGAACTCCTG
TIMP1_FW	CCTTCTGCAATTCCGACCT
TIMP1_RV	GCTTGGAACCCTTTATACATCTTG
ANGPT1_FW	AACCAGCCTCCTCTCTCA
ANGPT1_RV	GCAGCTGTATCTCAAGTCGAG
CYBB_FW	CTGATTCTCTTGCCAGTCTGT
CYBB_RV	ATTCCTGTCCAGTTGTCTTCG
HMOX1_FW	TCATGAGGAACTTTCAGAAGGG
HMOX1_RV	TGCGCTCAATCTCCTCCT
ITGB6_FW	GACTCCGGAAACATTCTCCAG
ITGB6_RV	CAAGTTGAGTCCTTCAGTGTCT
IL18_FW	CAGACCTTCCAGATCGCTTC
IL18_RV	AATTTCATTGCCACAAAGTTGATG
FGF9_FW	CAGGGAACCAGGAAAGACC
FGF9_RV	GGGTTAGTTTTTCTGATCCATACAG
MMP9_FW	CTTTGAGTCCGGTGGACGAT
MMP9_RV	TCGCCAGTACTTCCCATCCT
WNT5A_FW	CATGAACCTGCACAACAACG
WNT5A_RV	AGCCAGCATGTCTTCAGG

Supplementary Table 2- Rat Primers

Gene name	Primer Sequence
Cyclophilin_rFW	TCAACCCCACCGTGTTCTTC
Cyclophilin_rRV	TCCTTTCTCCCCAGTGCTCA
Anp_rFW	CCTGGACTGGGGAAGTCAAC
Anp_rRV	ATCTATCGGAGGGGTCCCAG
Col1A1_rFW	CCAGCCGCAAAGAGTCTACAT
Col1A1_rRV	AGGGACCCTTAGGCCATTGT
Tgfb1_rFW	GCTAATGGTGGACCGCAACA
Tgfb1_rRV	ACTGCTTCCCGAATGTCTGA
Fn1_rFW	CCCCTCCCAGAGAAGTGGT
Fn1_rRV	ATTGGGGTGTGGAAGGGTAAC
Tnc_rFW	GCAACCAGGGACAATGTGTG
Tnc_rRV	GGTACTCAGTGACCCGCATC
Ccl2_rFW	GCCTGTTGTTCACAGTTGCT
Ccl2_rRV	TGTAGTTCTCCAGCCGACTC
Il1B_rFW	GCACAGTTCCCCAACTGGTA
Il1B_rRV	TGTCCCGACCATTGCTGTTT
Foxm1_rFW	ACCAATATCCAGTGGCTTGG
Foxm1_rRV	GCTGTTGATCGCGAACTGTA
ENO3_rFW	CTCTACCGACACATTGCAGAC
ENO3_rRV	CCAACTTGTTTCCAGCATGAG
TIMP1_rFW	CAGCTTTCTGCAACTCGGA
TIMP1_rRV	ACAGCGTCGAATCCTTTGAG
ANGPT1_rFW	AGGAAACGAGAAGCAGAACTAC
ANGPT1__rRV	GTTGTCGTTATCAGCGTCCTT
CYBB_rFW	CTCAACCAGAATTCGAAGACAAC
CYBB__rRV	CCACTCCACGTTGAACAGAT
HMOX1_rFW	GCCTTCCTGCTCAACATTG
HMOX1_rRV	GCGAAGAAACTCTGTCTGTGA
ITGB6_rFW	TCACCCAAGAACAAGTCCATC
ITGB6_rRV	ACTTCTAGTTCCACCTCAGACC
IL18_rFW	CGGAGCATAAATGACCAAGTTC
IL18_rRV	GCCAGTCCTCTTACTTCACTATC
FGF9_rFW	TGGACAGTGGACTCTACCTC
FGF9_rRV	TTCTCTGAACACGCACTCC
MMP9_rFW	GAACTCACACAACGTCTTTCAC
MMP9_rRV	GGAGGTCATAGGTCACGTAGG
WNT5A_rFW	GCACGCATCCTCATGAACT
WNT5A_rRV	ATGGCACTTACAGGCTACATC
**Gene name**	**Primer Sequence**
rNID1_FW	GTGGAGCAGGATGGGTTCAA
rNID1_RV	TCCGTGTTGATGGTCAGGTG
rC1QTNF1_FW	CTTCCCCTCTGACCTAGCCT
rC1QTNF1_RV	TGTGCCAATCGAATCCTCCC
rSPARCL1_FW	CATGGAGCACTGCATAACGC
rSPARCL1_RV	GTGGTTAAAGCAAGCAGGGC
rMEGF9_FW	TTGCCAATGCAAACTGGGTG
rMEGF9_RV	ATCAGACCGGTTGTTGCACT
rCrtac1_FW4	GGCTGAGAGAGCCAGAGGA
rCrtac1_RV4	CCACTCCGTAGTTGAGCTGG

Supplementary Table 3- Antibodies

Antibodies	used WB Dilution	Catalog #	Company	Clone number
NID1 *	(1:500)	PA5-30103	ThermoFisher	Rabbit Polyclonal
MEGF9 *	(1:1000)	abx027366	Abbexa	Rabbit Polyclonal
CRTAC1	(1:1000)	ab254691	Abcam	Rabbit polyclonal
SPARCL1 *	(1:200)	sc-514275	Santa Cruz Biotechnology	(G-5): lot# A2615
C1QTNF1/CTRP1 *	(1:200)	sc-81943	Santa Cruz Biotechnology	(2E7): lot# K0222
NPPA/ANP	(1:200)	sc-515701	Santa Cruz Biotechnology	(F-2): lot# H0822
SPP1/OPN	(1:200)	sc-21742	Santa Cruz Biotechnology	(AKm2A1): lot# F1722
ITGA5	(1:500)	PA5-79529	ThermoFisher	Rabbit Polyclonal
ITGA10	(1:500)	PA5-67829	ThermoFisher	Rabbit Polyclonal
Vinculin (Validation)	(1:2000)	#4650	Cell Signaling	Rabbit Polyclonal
