# Supplementary material for: Transcriptional profiling unveils molecular subgroups of adaptive and maladaptive right ventricular remodeling in pulmonary hypertension
Source: Nat Cardiovasc Res. 2023 Sep 28;2(10):917–36. doi: 10.1038/s44161-023-00338-3 (PMC11358157; doi:10.1038/s44161-023-00338-3)
Supplement: Supplementary file 2 — Reporting Summary [file 44161_2023_338_MOESM2_ESM.pdf]

nature portfolio

Corresponding author(s): Soni Pullamsetti

Last updated by author(s): Aug 18, 2023

Reporting Summary

Nature Portfolio wishes to improve the reproducibility of the work that we publish. This form provides structure for consistency and transparency in reporting. For further information on Nature Portfolio policies, see our Editorial Policies and the Editorial Policy Checklist.

Statistics

For all statistical analyses, confirm that the following items are present in the figure legend, table legend, main text, or Methods section.

n/a	Confirmed	
☐	☒	The exact sample size (*n*) for each experimental group/condition, given as a discrete number and unit of measurement
☐	☒	A statement on whether measurements were taken from distinct samples or whether the same sample was measured repeatedly
☐	☒	The statistical test(s) used AND whether they are one- or two-sided<br>*Only common tests should be described solely by name; describe more complex techniques in the Methods section.*
☐	☒	A description of all covariates tested
☐	☒	A description of any assumptions or corrections, such as tests of normality and adjustment for multiple comparisons
☐	☒	A full description of the statistical parameters including central tendency (e.g. means) or other basic estimates (e.g. regression coefficient) AND variation (e.g. standard deviation) or associated estimates of uncertainty (e.g. confidence intervals)
☐	☒	For null hypothesis testing, the test statistic (e.g. *F*, *t*, *r*) with confidence intervals, effect sizes, degrees of freedom and *P* value noted<br>*Give P values as exact values whenever suitable.*
☒	☐	For Bayesian analysis, information on the choice of priors and Markov chain Monte Carlo settings
☐	☒	For hierarchical and complex designs, identification of the appropriate level for tests and full reporting of outcomes
☐	☒	Estimates of effect sizes (e.g. Cohen's *d*, Pearson's *r*), indicating how they were calculated

Our web collection on statistics for biologists contains articles on many of the points above.

Software and code

Policy information about availability of computer code

Data collection	RV and RNA sample collection are described in detail in the method and supplementary information files of the revised manuscript
Data analysis	RNA-seq and plasma proteome data analysis are described in detail in the method and supplementary information files of the revised manuscript

For manuscripts utilizing custom algorithms or software that are central to the research but not yet described in published literature, software must be made available to editors and reviewers. We strongly encourage code deposition in a community repository (e.g. GitHub). See the Nature Portfolio guidelines for submitting code & software for further information.

Data

Policy information about availability of data

All manuscripts must include a data availability statement. This statement should provide the following information, where applicable:

- Accession codes, unique identifiers, or web links for publicly available datasets
- A description of any restrictions on data availability
- For clinical datasets or third party data, please ensure that the statement adheres to our policy

Gene expression profiling data are already deposited in gene expression omnibus repository (GEO) from NCBI. The accession number is provided in paper and the repository will be public after publication. The clinical information of subjects from whom human RV and blood samples were obtained are provided as Source data and Supplementary datasets. Data supporting the findings of this study are available within the article and its supplementary information files.

Human research participants

Policy information about studies involving human research participants and Sex and Gender in Research.

Reporting on sex and gender	The information regarding the sex of human subjects in transcriptome and two proteome cohort has been provided in Source data 3, 6, 17, and 18.
Population characteristics	All demographic and clinical characteristics of patients involved in this study are extensively described within the Source data 3, 17, and 18.
Recruitment	Human RV tissue collection for RNA-seq datasets: Tissues were obtained from patients who had previously given written, informed consent. Patients were classified as control, or with compensated, or decompensated RV condition, based on clinical history and cardiac index. The procedures and criteria for the acquisition of control, compensated, and decompensated RV sampleswent as previously described (reference 22), and detailed information is provided in Human RV tissue collection in methods and Source data 3.<br><br>PAH plasma discovery cohort, has been performed with the approval of the local ethics committee (AZ 58/15) at University Hospital Giessen and Marburg, Department of Pneumology and Critical Care Medicine, Germany from 2016 to 2018. Patients in this study have signed an informed consent before the sample collection. Blood samples from 35 IPAH patients (male and female) participating in the "Right Heart 1 trial" (NCT03403868) were collected at University Hospital Giessen and Marburg, Department of Pneumology and Critical Care Medicine. Fasting blood sample were obtained during right heart catheterization and immediately frozen at -80 degree for the following proteomic assay. All the clinical information of the patients (including age) has been provided in Source data. 17. Discovery cohort does not contain any control group. In brief, PAH patients from discovery cohort has been characterized using coupling ratio of Ees/Ea as previously described.<br><br>UK cohort plasma proteome has been done with the approval of Laval University and the IUCPQ Biosafety and Ethics Committees (CER#20735), and the University of Sheffield from the Sheffield Teaching Hospitals Observational Study of Patients with Pulmonary Hypertension, Cardiovascular and Lung Disease (UK REC Ref 18/YH/0441) from 2013 to 2018. All patients gave informed consent to be part of the study beforehand. Blood samples 61 PAH patients (male and female) undergoing right heart catheterization (RHC) has been obtained at Sheffield Pulmonary Vascular Unit (UK) from March 2013 to February 2018, with support from the NIHR Sheffield Clinical Research Facility. Fasting blood samples were collected following RHC measurements in both cohorts of study, and immediately frozen at -80 degree until proteomics analysis. All the clinical information of the patients (including age) has been provided in Source data. 18. Similar to the transcriptome cohort, subjects who were not diagnosed for any cardiac or respiratory diseases were selected as control group in the validation proteome cohort. Primarily characterization has been performed based on Cardiac Index value, as compensated RV identified with CI > 2.2 L/min/m2 and decompensated RV with CI < 2.2 L/min/m2.<br><br>Normal distribution of age and gender per group of patients (compensated/decompensated) has been tested in all cohorts using Welch two-sample T-test for age, and Fisher's exact test for Sex, to confirm that there is no significant effects of these two factors in any of the comparisons. (Source data 17, 18)
Ethics oversight	Human RV tissue collection for RNA-seq datasets: the experimental procedures for using human tissues or cells conformed to the principles outlined in the Declaration of Helsinki, and has been performed with the approval of Laval University and the Biosafety and Ethics committees of the University Institute of Cardiology and Respirology of Quebec (CER#20773, CER#20735, CER#21747). All experiments were performed in accordance with the latest preclinical PAH research guidelines.<br><br>Preclinical PAH experiments in rats were performed according to the guidelines of the Canadian Council on Animal Care and approved by the Animal Care and Use committees of Laval University (2014-176 and 2018-015).<br><br>Discovery Cohort: Studies on PAH plasma have been performed with the approval of the local ethics committee (AZ 58/15) at University Hospital Giessen and Marburg, Department of Pneumology and Critical Care Medicine, Germany from 2016 to 2018 for German cohort. Patients in this study have signed an informed consent before the sample collection.<br><br>UK cohort: All experimental procedures have been done with the approval of Laval University and the IUCPQ Biosafety and Ethics Committees (CER#20735), and the University of Sheffield from the Sheffield Teaching Hospitals Observational Study of Patients with Pulmonary Hypertension, Cardiovascular and Lung Disease (UK REC Ref 18/YH/0441) from 2013 to 2018. All patients gave informed consent to be part of the study beforehand.

Note that full information on the approval of the study protocol must also be provided in the manuscript.

Field-specific reporting

Please select the one below that is the best fit for your research. If you are not sure, read the appropriate sections before making your selection.

☒ Life sciences ☐ Behavioural & social sciences ☐ Ecological, evolutionary & environmental sciences

For a reference copy of the document with all sections, see nature.com/documents/nr-reporting-summary-flat.pdf

Life sciences study design

All studies must disclose on these points even when the disclosure is negative.

Sample size	The sample size of human biopsies and rat right ventricles used were up to 60mg based on the availability of the samples.<br>Human RNA-seq was performed on 40 human RV tissues that were clinically classified Into: 13 control, 14 compensated RV, and 13 decompensated RV states. (Source data 3)<br><br>Transcriptomic profiling in first MCT rats was performed on RV tissues obtained from 30 samples: 10 control, 10 compensated, and 10 decompensated RVs, (3 compensated and 1 decompensated samples have been removed in downstream analysis)<br>Second MCT batch was performed for 29 RV samples: 8 control, 12 compensated and 9 decompenstated RV, (1 compensated and 1 decompensated have been removed in downstream analysis)<br>PAB RNAseq was performed for 15 RV samples: 5 control, 5 compensated and 5 decompensated RV. (1 decompensated has been removed for downstream analysis) (Source data 1, 2, and 8)<br><br>German Cohort (discovery): Blood samples from 35 IPAH patients were collected (Germany) from 2016 to 2018 . 20 compensated and 15 decompensated RV patients. (Source data 17)<br>UK cohort (served as validation): Blood samples 61 PAH patients has been obtained at Sheffield Pulmonary Vascular Unit (UK) from March 2013 to February 2018. (56 control, 26 compensated and 35 decompensated RV patients)
Data exclusions	4 samples of first MCT and 2 samples from second MCT , 1 sample from PAB animal model has been removed. (Supplementary Table 1,3,9)<br>From human transcriptome one sample has been removed due to the sequencing error and four other samples has been removed as outliers. (Source data 3)<br>No sample has been removed from the plasma cohorts. (Source data 17, 18)
Replication	All biological replicates (from human biopsies, rat ventricles, and blood samples from both cohorts) are reported in the respective figure legends and methods sections of this manuscript.
Randomization	N/A
Blinding	All omics analysis, including RNA-sequencing and olink were performed blinded (unbiased). Hemodynamic analysis, immunohistochemical, PCR and Western blot anaylsis were all blinded during data collection and analysis.

Reporting for specific materials, systems and methods

We require information from authors about some types of materials, experimental systems and methods used in many studies. Here, indicate whether each material, system or method listed is relevant to your study. If you are not sure if a list item applies to your research, read the appropriate section before selecting a response.

Materials & experimental systems

n/a	Involved in the study	
☐	☒	Antibodies
☒	☐	Eukaryotic cell lines
☒	☐	Palaeontology and archaeology
☐	☒	Animals and other organisms
☒	☐	Clinical data
☒	☐	Dual use research of concern

Methods

n/a	Involved in the study	
☒	☐	ChIP-seq
☒	☐	Flow cytometry
☒	☐	MRI-based neuroimaging

Antibodies

Antibodies used	#Antibodies #WB Dilution #Catalog #Company #Clone_number<br>NID1 (1:500) PA5-30103 ThermoFisher Rabbit Polyclonal<br>MEGF9 (1:1000) abx027366 Abbexa Rabbit Polyclonal<br>CRTAC1 (1:1000) ab254691 Abcam Rabbit polyclonal<br>SPARCL1 (1:200) sc-514275 Santa Cruz Biotechnology (G-5): lot# A2615<br>C1QTNF1/CTRP1 (1:200) sc-81943 Santa Cruz Biotechnology (2E7): lot# K0222<br>NPPA/ANP (1:200) sc-515701 Santa Cruz Biotechnology (F-2): lot# H0822<br>SPP1/OPN (1:200) sc-21742 Santa Cruz Biotechnology (AKm2A1): lot# F1722<br>ITGA5 (1:500) PA5-79529 ThermoFisher Rabbit Polyclonal<br>ITGA10 (1:500) PA5-67829 ThermoFisher Rabbit Polyclonal<br>Vinculin (Validation) (1:2000) #4650 Cell Signaling Rabbit Polyclonal<br>(Supplementary dataset 4)
Validation	Vinculin (cellsignaling #4650)

Animals and other research organisms

Policy information about studies involving animals; ARRIVE guidelines recommended for reporting animal research, and Sex and Gender in Research

Laboratory animals	Male and female Sprague Dawley rats (Rattus norvegicus) at age 8-12 weeks, (Charles River Laboratories, Wilmington, MA, USA) were subjected to the treatment protocol as previously described (22). In brief, For MCT model, a single subcutaneous injection of MCT (60 mg/kg) has been applied, (Control rats received saline), and the RV function was monitored weekly using echocardiography. Terminal right-heart catheterization (RHC) was performed on anesthetized, closed-chest rats. The whole experiment procedure was 5 weeks for MCT rat model. For PAB model, following anesthesia, the PA was separated from the aorta and left atrium, and was tied against a 19-gauge needle and then released quickly. PAB-operated rats with peak velocities > 3.5 m/s at the banding site (assessed by echocardiography), as well as sham-operated rats without tying the pulmonary trunk were included for the study. Between weeks 3 and 8 following PAB operation, sham and PAB-operated rats were sacrificed or euthanatized following the Echocardiography, and underwent terminal right-heart catheterization (RHC) at the end of protocol, based on clinical symptoms of RV failure. (Source data 1, 2, 8)
Wild animals	None
Reporting on sex	The central objective of this study was to classify the pathophysiology of RV function into normal, compensated, and decompensated states, based on hemodynamic parameters. As based on previous studies and assessments in this study, only males exhibit an aggressive course of MCT-induced pulmonary hypertension compared to females, (PMID: 23821593), we mainly used male rats subjected to the treatment protocol (PAB or MCT) pulmonary hypertension. MCT-induced female rats has been used to compare the molecular phenotype with male rats and the results are reported mainly in Figure 4.
Field-collected samples	None
Ethics oversight	Preclinical PAH experiments in rats were performed according to the guidelines of the Canadian Council on Animal Care and approved by the Animal Care and Use committees of Laval University (2014-176 and 2018-015).

Note that full information on the approval of the study protocol must also be provided in the manuscript.
