# Supplementary figures and images for: Transcriptional profiling unveils molecular subgroups of adaptive and maladaptive right ventricular remodeling in pulmonary hypertension
Source: Nat Cardiovasc Res. 2023 Sep 28;2(10):917–36. doi: 10.1038/s44161-023-00338-3 (PMC11358157; doi:10.1038/s44161-023-00338-3)

Uncropped scan of Gel 1: Extended data 4b

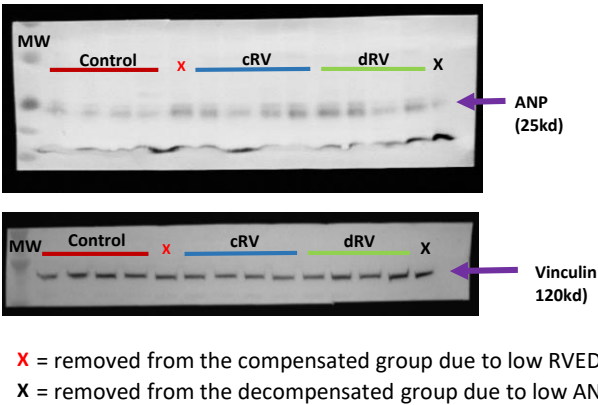

Uncropped scan of Gel 2: Extended data 4b

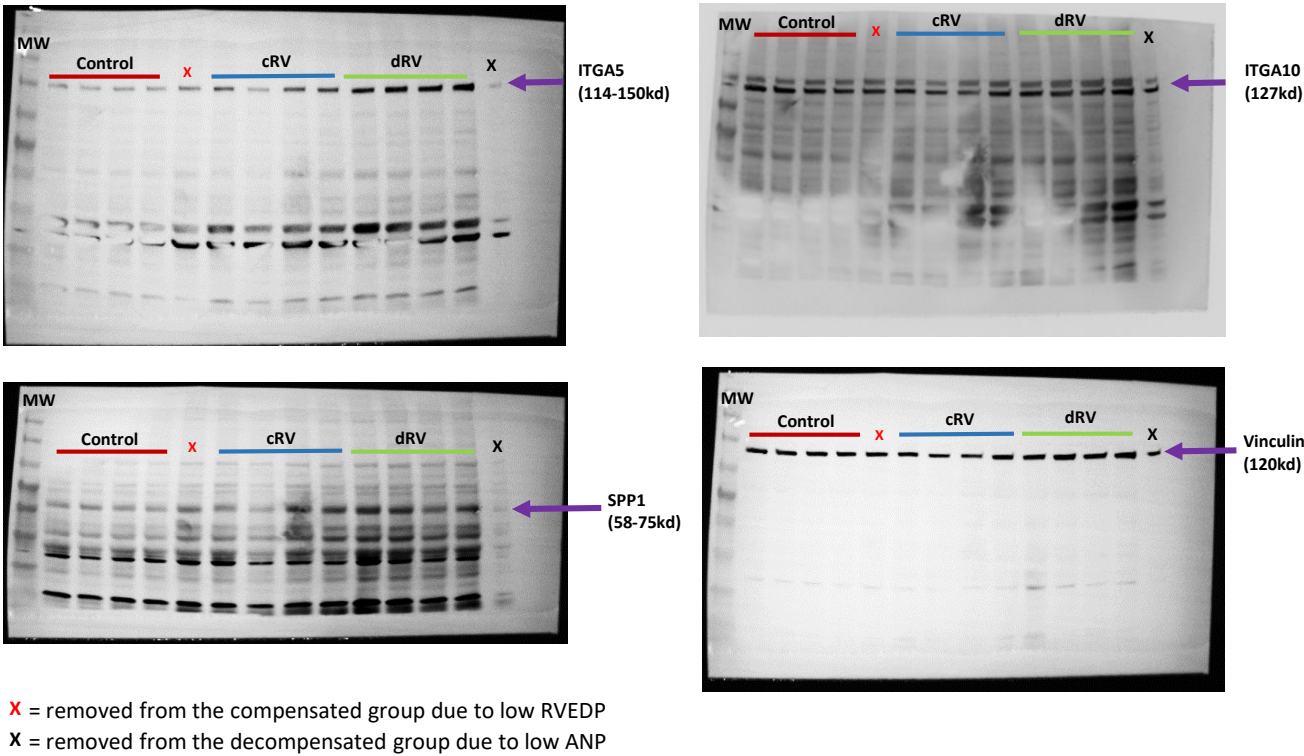

Supplement: Supplementary file 13 — Uncropped scan of western blot gels 1 and 2. [file 44161_2023_338_MOESM13_ESM.pdf]

Uncropped scan of Gel 1: Extended data 7c

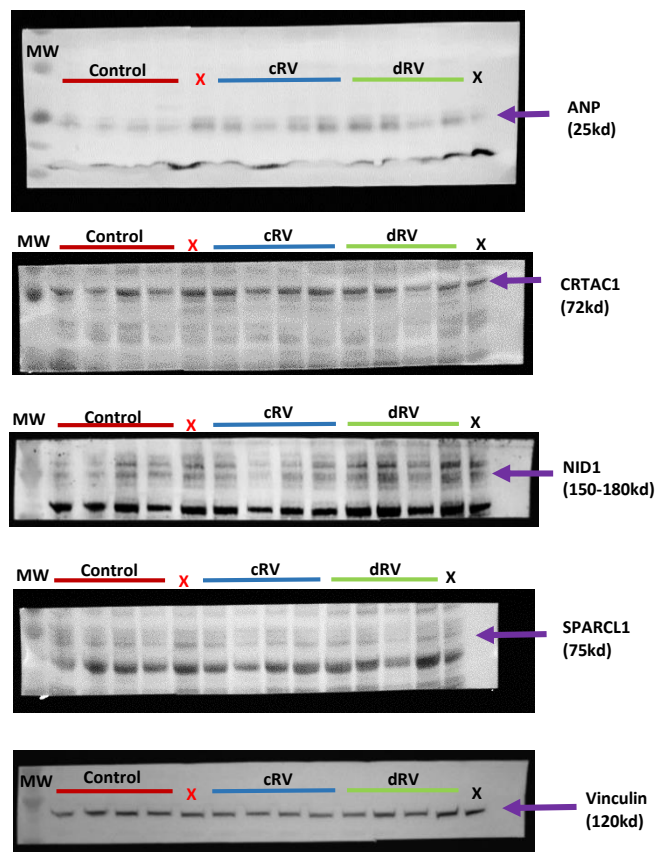

Uncropped scan of Gel 3: Extended data 7c

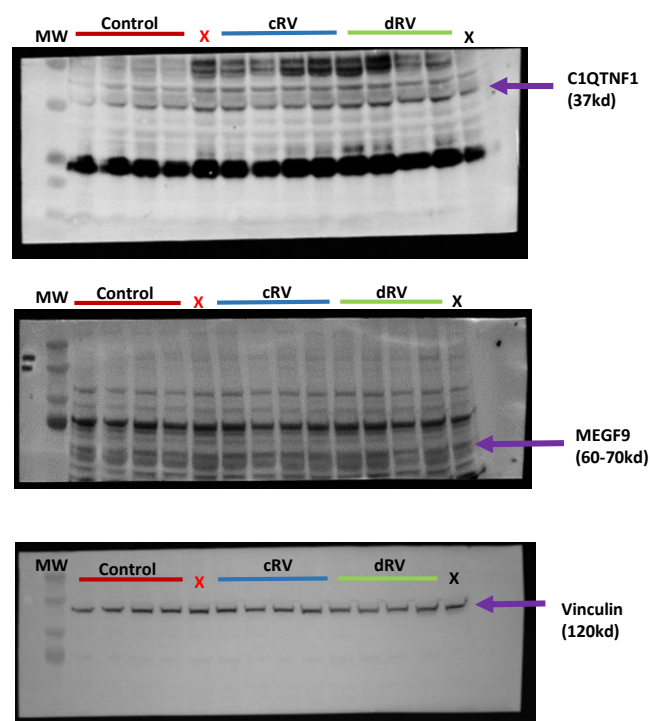

Supplement: Supplementary file 17 — Uncropped scan of western blot gels 1 and 3. [file 44161_2023_338_MOESM17_ESM.pdf]
